# Altered gene expression in human brain microvascular endothelial cells in response to the infection of influenza H1N1 virus

**DOI:** 10.1186/s44149-022-00053-9

**Published:** 2022-11-03

**Authors:** Doaa Higazy, Xianwu Lin, Tanghui Xie, Ke Wang, Xiaochen Gao, Min Cui

**Affiliations:** 1 Microbiology Department, Faculty of Agriculture, Cairo University, Giza, 12613 Egypt; 2 Key Laboratory of Preventive Veterinary Medicine in Hubei Province, The Cooperative Innovation Center for Sustainable Pig Production, Wuhan, 430070 Hubei China; 3 Key Laboratory of Development of Veterinary Diagnostic Products, Ministry of Agriculture of the People’s Republic of China, Wuhan, 430070 Hubei China; 4 International Research Center for Animal Disease, Ministry of Science and Technology of the People’s Republic of China, Wuhan, 430070 Hubei China; 5 State Key Laboratory of Agricultural Microbiology, College of Veterinary Medicine, Huazhong Agricultural University, No.1 Shizishan St. Huazhong Agricultural University, Wuhan, 430070 Hubei China; 6 College of Informatics, Huazhong Agricultural University, Wuhan, 430070 Hubei China

**Keywords:** Blood–brain barrier, Influenza A virus (IAV), hBMECs, CNS, Neurodegenerative diseases, RNAseq

## Abstract

Influenza viruses not only cause respiratory illness, but also have been reported to elicit neurological manifestations following acute viral infection. The central nervous system (CNS) has a specific defense mechanism against pathogens structured by cerebral microvasculature lined with brain endothelial cells to form the blood–brain barrier (BBB). To investigate the response of human brain microvascular endothelial cells (hBMECs) to the Influenza A virus (IAV), we inoculated the cells with the A/WSN/33 (H1N1) virus. We then conducted an RNAseq experiment to determine the changes in gene expression levels and the activated disease pathways following infection. The analysis revealed an effective activation of the innate immune defense by inducing the pattern recognition receptors (PRRs). Along with the production of proinflammatory cytokines, we detected an upregulation of interferons and interferon-stimulated genes, such as IFN-β/λ, ISG15, CXCL11, CXCL3 and IL-6, etc. Moreover, infected hBMECs exhibited a disruption in the cytoskeletal structure both on the transcriptomic and cytological levels. The RNAseq analysis showed different pathways and candidate genes associated with the neuroactive ligand-receptor interaction, neuroinflammation, and neurodegenerative diseases, together with a predicted activation of the neuroglia. Likewise, some genes linked with the mitochondrial structure and function displayed a significantly altered expression. En masse, this data supports that hBMECs could be infected by the IAV, which induces the innate and inflammatory immune response. The results suggest that the influenza virus infection could potentially induce a subsequent aggravation of neurological disorders.

**Supplementary Information:**

The online version contains supplementary material available at 10.1186/s44149-022-00053-9.

## Introduction

The blood–brain barrier precisely controls the central nervous system (CNS) to protect neurons from the external passage of pathogens and toxins into the brain (Daneman and Prat 2015; Nassif et al. 2002). The brain microvascular endothelial cells (BMVECs) are remarkable constituents of the BBB and provide selective permeability (Rosas-Hernandez et al. 2018). Pathogens, including viruses, can damage the brain's microvascular structure and lead to the dysfunction and hyperpermeability of the BBB, causing numerous migrations of immune cells to the brain and resulting in an inflammation that could trigger several neurological disorders (Koyuncu et al. 2013). There is an association between peripheral and central inflammation by the passage of injury signals to the brain that initiates cytokine production or blood-borne mediators crossing to anatomically sensitive sites within the BBB (Anthony et al. 2012; Lampa et al. 2012). The respiratory system is not the only route targeted by the Influenza A virus (IAV). It is demonstrated that the virus could infect the CNS and cause neuronal disorders (Studahl 2003; van Riel et al. 2014).

Previous investigations have confirmed that influenza A and B viruses are linked to the incidence of mild encephalopathy with the reversible splenial lesion (MERS) (Vanderschueren et al. 2018). MERS is a common cause of reversible lesions involved in the splenium of corpus callosum (Garcia-Monco et al. 2011). In one case report, MERS was a complication of influenza B primary infection for an 8-year-old girl who was not previously vaccinated (Ventresca et al. 2021). They notably observed an acute lesion in the splenium of the corpus callosum. The lesion was transient, which suggested that the virus's effect on the brain was reversible (Ventresca et al. 2021). Another case was reported for a 4-year-old healthy female child who suffered from influenza-associated encephalopathy (IAE); the visualized symptoms included neurological complications of temporary visual impairment and significant motor deficits (Billa et al. 2020).

However, the mechanism by which the influenza virus could induce neuroinflammation is not fully understood. Certain influenza virus strains, including A/WSN/33, have been classified as neurotropic since the viral vRNA and mRNA were detected in the brain by real-time PCR following olfactory infection of mice (Aronsson et al. 2001). Neurovirulent strains can cross the CNS through the olfactory, vagus, trigeminal, and sympathetic nerves (Park et al. 2002). On the other hand, non-neurotropic strains such as A/PR/8/34 could induce cognitive deterioration (Jurgens et al. 2012). In this scenario, the activated immune response can decrease the neurotrophic (BDNF, NGF) and immunomodulatory (CD200, CXCL1) factors within the hippocampus while increasing the microglial reactivity (Jurgens et al. 2012). Therefore, neurotropic and non-neurotropic IAV strains might harm the CNS (Barbosa-Silva et al. 2018). Following influenza virus infection, the astrocytes induce the flow of several proinflammatory cytokines in addition to the upregulation of genes functioning in synaptic transmission (Lin et al. 2015). IAV might aggravate multiple sclerosis coincident with CXCL5 upregulation following peripheral infection (Blackmore et al. 2017). In parallel, the increased passage of monocytes and neutrophils into the brain enhances the transcriptomic changes of the spinal cord and cerebellum (Blackmore et al. 2017). Furthermore, H7N7 and H3N2 could cause spine loss in the hippocampus, a slow recovery following infection, and long-term damage to the CNS (Hosseini et al. 2018).

H5N1 causes similar pathological aspects to Parkinsonism, including loss of dopaminergic phenotype in substantia nigra pars compacta (SNpc) and alterations in the number and morphology of SNpc microglia (Jang et al. 2012; Rohn and Catlin 2011). In one study, B and T cells deficient mice inoculated with H1N1 in the brainstem, and hypothalamic neurons appeared to suffer from potential per se narcoleptic-like sleep disruption (Tesoriero et al. 2016). Further investigations on chickens infected with the highly pathogenic influenza virus H7N1 suggested that the virus infected the brain endothelial cells at the early stages of 24 hpi, which subsequently disrupted the tight junctions of BBB and caused virus leakage into adjacent neuroparenchyma (Chaves et al. 2014). To our knowledge, this is the first research to study the hBMECs exposed to A/WSN/1933 (H1N1) influenza virus strain. Here, we found that the human brain microvascular endothelial cells (hBMECs) are susceptible to IAVs A/WSN/33 (H1N1). The infection was accompanied by a massive alteration in gene expression associated with producing several IFN genes and activating neuroinflammation signaling pathways. The neuroactive ligand-receptor interaction pathway was significantly up-regulated, coinciding with the induced disruption in cell cytoskeleton and mitochondrial dysfunction on the transcriptomic level.

## Results

### Influenza A Virus Invades hBMECs

**Fig. 1:**
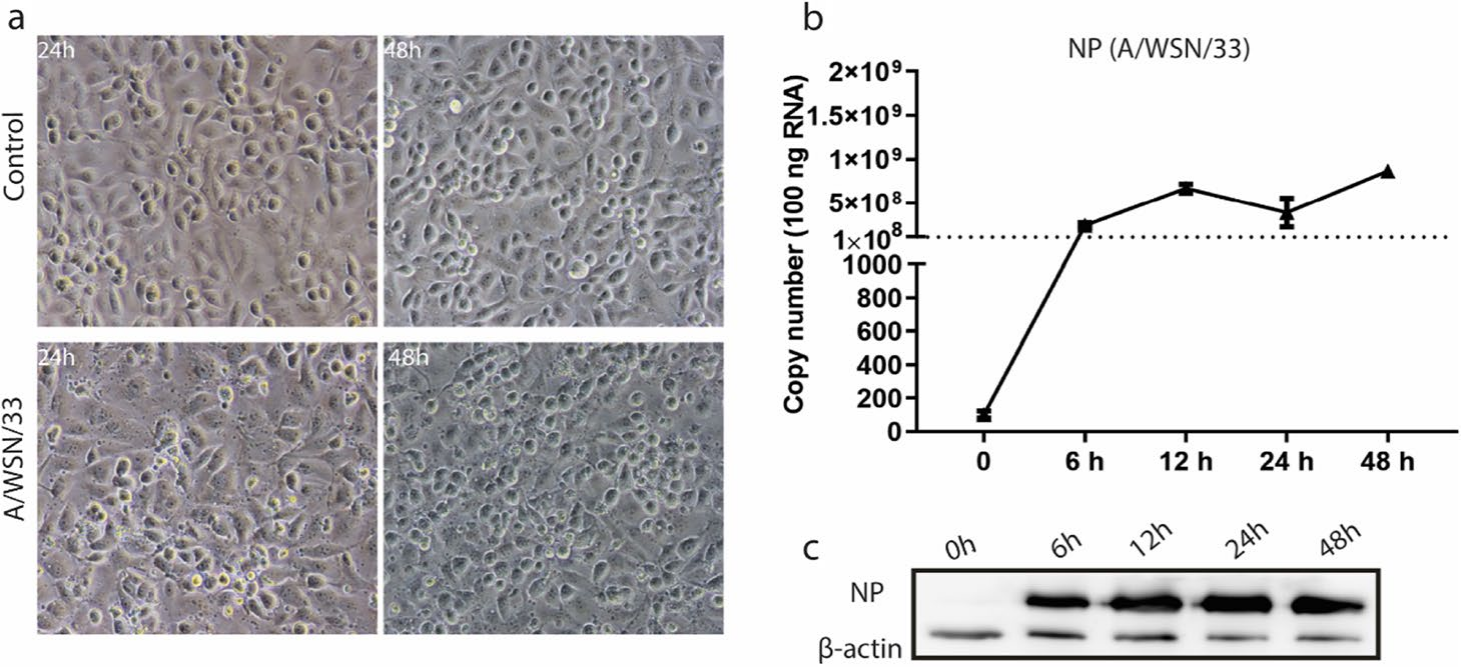
Influenza virus entry and infection to human brain microvascular endothelial cells (hBMECs). **a** Phase-contrast (Ph) microscopy visualization of hBMECs morphology infected by A/WSN/33 (H1N1) at 0.1 multiplicity of infection (MOI). **b** RNA extracted from hBMECs and targeted by RT-qPCR to detect NP gene expression at different time points, error bars indicate standard deviation. **c** the cells were lysed for NP detection by western blotting at indicated time points post-infection. Data were shown as means ± SEM from three experiments, and statistical significance analyzed by t-test * *p* < 0.05, *** *p* < 0.001

To assess the infection of A/WSN/33 in hBMECs, we inoculated the cultured cells with IAV A/WSN/33 strain at multiplicity of infection (MOI) 0.1. Morphological changes were monitored with Ph- microscopy. The cytopathic effect became dramatically obvious at 24 hpi (h post infection), and 48 hpi (Fig. 1a). The IAV nucleoprotein (*NP*) gene expression was detected over different time points of infection by RT-qPCR that significantly raised at 6 hpi and continued to increase till 48 hpi (Fig. 1b). We collected the supernatant to determine the virus titer which was around 10^2^ ~ 10^3^ (data not shown). Simultaneously, the translated nucleoprotein was recognized by western blot shortly at 6 hpi and showed high protein intensities at 24 hpi, and 48 hpi (Fig. 1c).

**Fig. 2:**
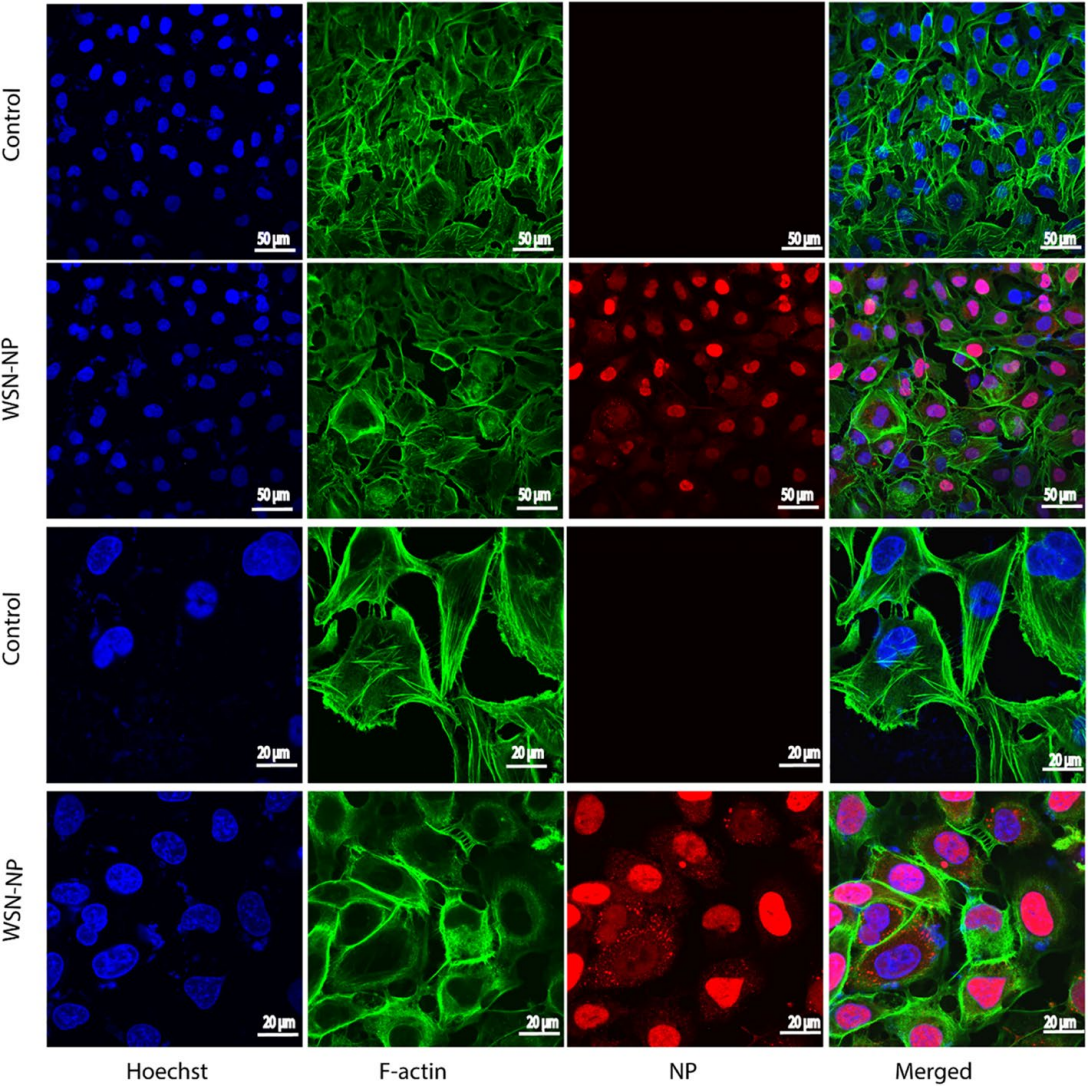
Immunoflourence for human brain microvascular endothelial cells (hBMECs) at 12 hpi with Influenza A virus. hBMECs were mock-treated or infected with A/WSN/33 (H1N1) virus at 0.1 MOI. After 12 h, the cells were stained with anti-NP (red), Phalloidin for F-actin (green), and Hoechst for nucleic acid stain (Blue). Images were captured by confocal microscopy. The column “Merged” is generated by the machine software, which is produced by positioning the “red”, “green” and “blue” fluorescence of the same cells within the same optical plane: scale bar 50, 20 µM

The influenza virus can shuttle between the nucleus and cytoplasm. Hence, the pivotal role of the influenza virus NP is to encapsidate the virus genome for transcription, replication, and packaging, in addition to its ability to interact with cellular polypeptides, including actin (Portela and Digard 2002; Terrier et al. 2016). The hBMECs were seeded in a 24-well plate and infected with A/WSN/33 at 0.1 MOI. After 12 h, the cells were fixed, permeabilized, and blocked. The cells were stained for the anti-NP (red), F-actin (green), and nucleic acids (blue) and further visualized by laser confocal microscopy (Fig. 2). The results indicated that H1N1 could hijack into hBMECs and find its way to the cell nucleus. In contrast, the intact cells showed no signal to the virus NP. Briefly, the virus NP appeared to accumulate in the cell nucleus and to slightly colocalize with actin filaments. The filamentous actin cytoskeleton showed a re-organization and disruption within infected cells. It appeared to be thicker and displayed an irregular doughnut shape compared to the intact cells. We also noticed a granule-shaped virus NP in the cytoplasm and near the edges of the plasma membrane. These data demonstrated that hBMECs are highly susceptible to IAV invasion.

### Altered Gene Expression in Infected hBMECs

**Fig. 3:**
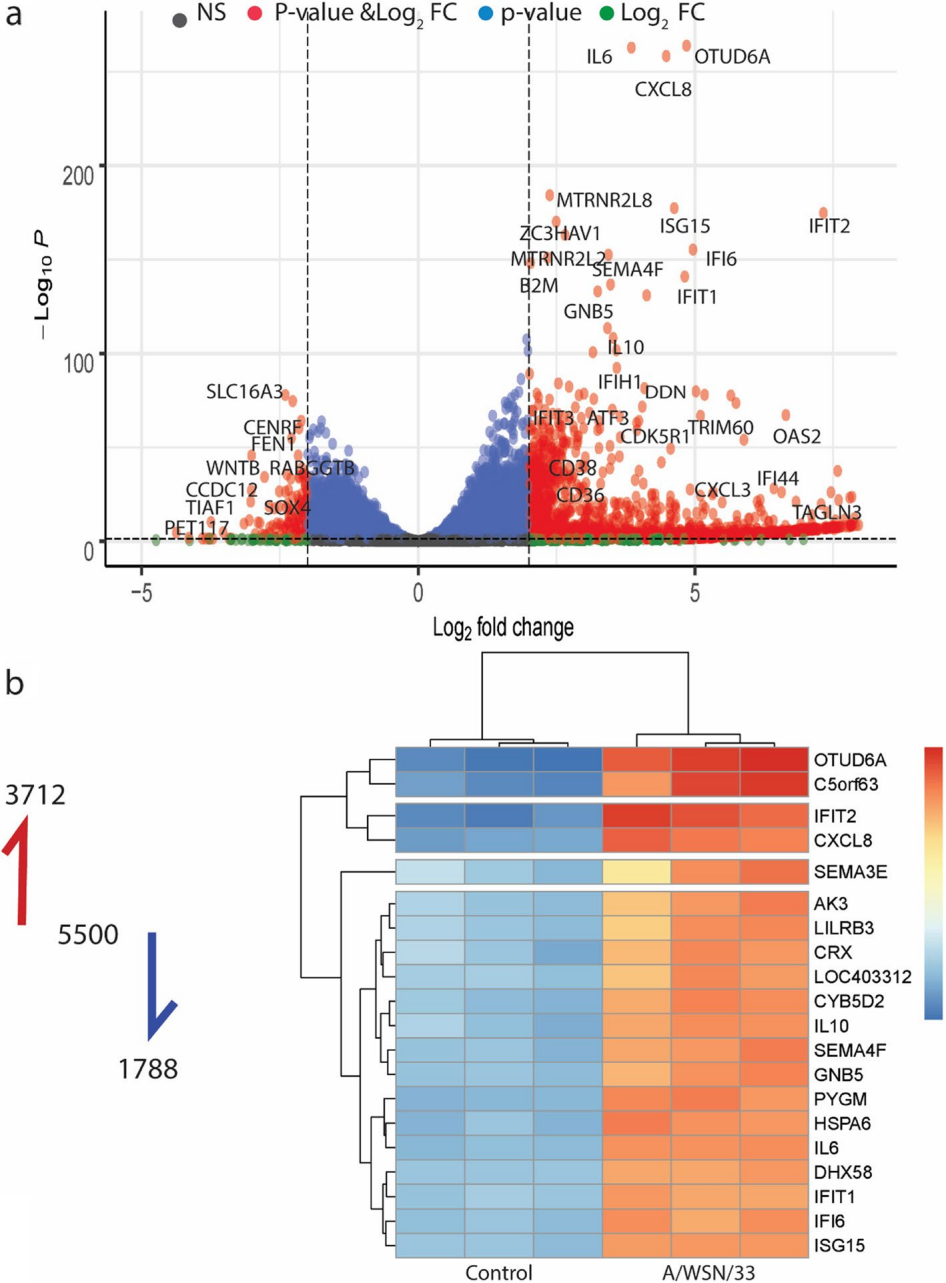
Transcriptomic analysis of human brain microvascular endothelial cells (hBMECs) following infection with Influenza A virus. **a** Volcano plot of host genes differentially expressed at 12 hpi (padj < 0.05) and ± 2 log2FC change. Each dot represents a gene. The red, blue, green, and black dots represent the differentially expressed genes within the selected *p*-value (padj < 0.05) and log2FC ± 2, the *p*-value, the log2FC, and non-significant genes, respectively. **b** Heatmap of the top 20 genes induced in hBMECs treated with the A/WSN/33 compared to the control cells at 12 hpi (padj < 0.05), the genes are listed on the Y-axis. Each column represents one sample, the control cells are represented in three columns (replicates), and the same case is for the treatment. The overall number of differentially expressed genes is 5,500, among which 3,712 were up-regulated and 1,788 were down-regulated (|log2FC|≥ 1; padj < 0.05) A list of the differentially expressed genes is available in Supplementary file 2

We performed a total RNA extraction at 12 h following the inoculation with A/WSN/33 (H1N1). The cDNA library was constructed and then sequenced by the BGISEQ-500 platform. The data were mapped, assembled, and annotated by HISAT2/StringTie and DESeq2. Outwardly, the differentially expressed genes (DEGs) proclaimed a significant alteration for the mRNA of the infected hBMECs compared to the non-infected cells. The analysis listed a total of 5,500 differentially expressed genes, among which 3,712 were up-regulated, and 1,788 were down-regulated (|log2FC|≥ 1; padj < 0.05), the DEGs of infected hBMECs are available and listed in Supplementary file 2. The volcano plot (Fig. 3a) displayed the genes of infected hBMECs at 12 hpi with (|log2FC|≥ 2; padj < 0.05). The heatmap (Fig. 3b) pointed out the top 20 significant genes expressed in infected hBMECs compared to intact cells. Interestingly, genes of antiviral activity were induced in parallel to the interferon stimulating genes (*ISGs*), including interferon-induced proteins with tetratricopeptide repeats (*IFITs*) and interferon-induced proteins such as *IFI44* that were all significantly up-regulated following H1N1 infection. Several regulatory genes such as *DHX58* (the Probable ATP-dependent RNA helicase), *ISG15* (Interferon-stimulated gene 15), *IRF7* (Interferon regulatory factor 7) and *TRIM15* (Tripartite Motif Containing 15) were associated with type I interferon production also increased dramatically. On the other hand, type III interferon genes *IFN-λ1*, *IFN-λ2*, and *IFN-λ3* were highly up-regulated in gene expression than type I interferon *IFN-α* and *IFN-β*, along with other up-regulated cytokines, including *CXCL2*, *CXCL3*, *CXCL8*, *CXCL10*, *CXCL11* and *CXCL16*. The results indicated a significant alteration in the mRNA level for hBMECs infected with A/WSN/33.

### Enrichment analysis predicts enhanced disruption of the cells’ cytoskeletal structure

**Fig. 4:**
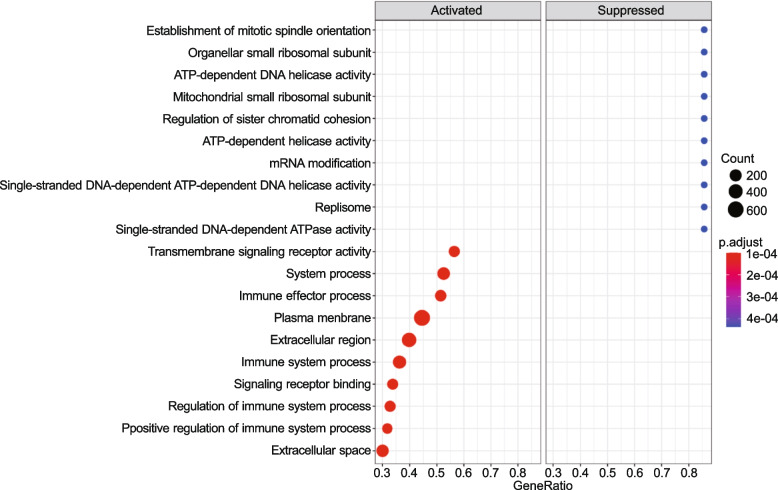
Functional enrichment analysis of the Differentially expressed genes in human brain microvascular endothelial cells (hBMECs) at 12 hpi. Gene ontology terms plotted in the order of gene ratio, the size of the dots depicts the number of the gene counts that were significantly enriched in the GO list, the dot color represents the *p*-adjusted value (padj ≤ 0.05)

**Fig. 5:**
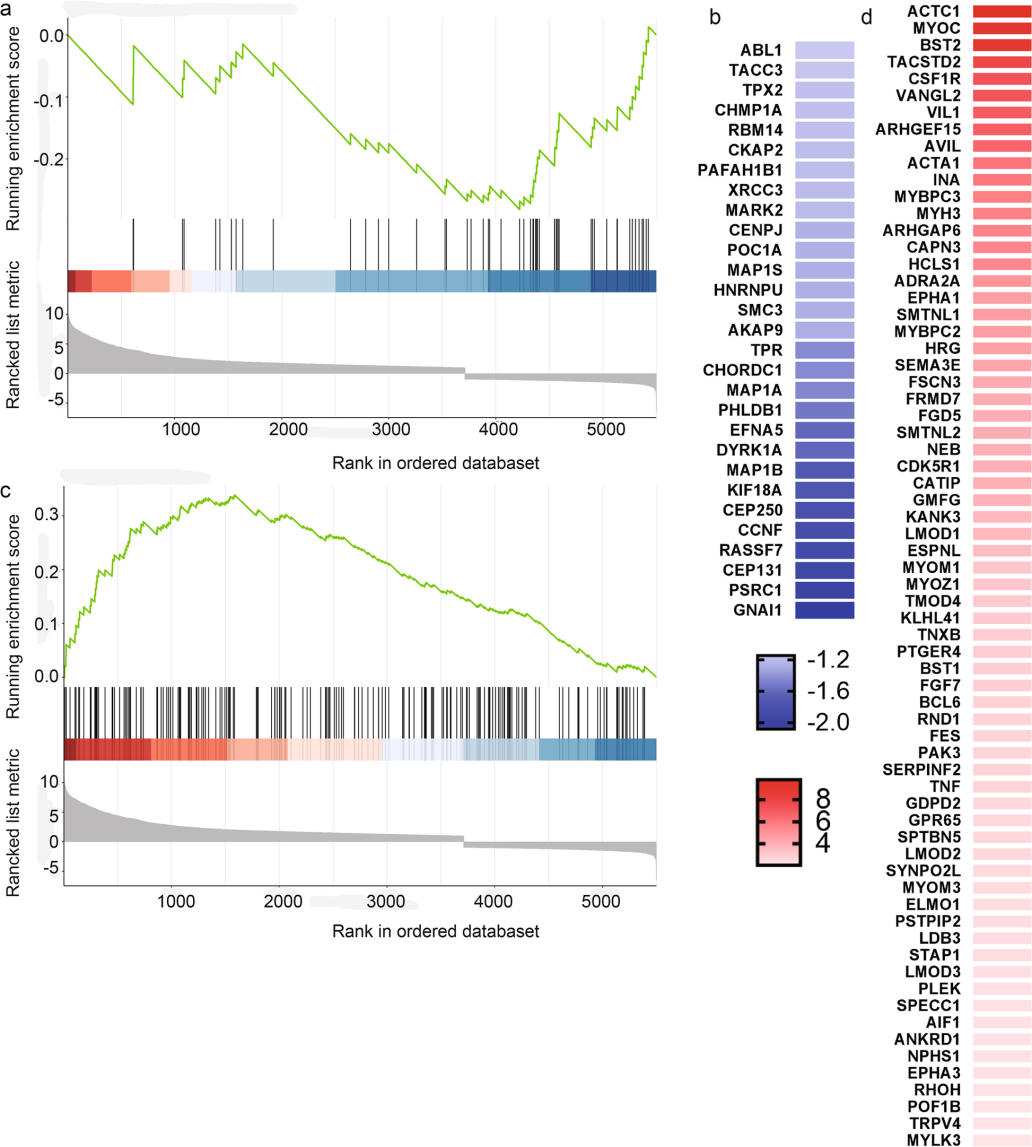
GSEA plots for enriched genes regulating the cytoskeleton structure. **a** GSEA plot indicating the negative running enrichment score for the “regulation of microtubule cytoskeleton organization” (NES = -2.3, padj = 0.01) (**b**) with a number of the down-regulated genes participating in the process displayed in the form of a heatmap. **c** GSEA plot showing the positive running enrichment score activating the “actin cytoskeleton organization” (NES = 1.31, padj = 0.02) (**d**) with a list of the top activated genes shown in a heatmap. Black bars underneath the graph present the rank positions of genes from the gene set. The green line refers to the enrichment profile. GSEA, gene set enrichment analysis

The R package clusterProfiler identified the GO and KEGG enriched terms based on gene set enrichment analysis, while the network analysis and canonical pathways were generated by IPA. The GO-based gene set enrichment analysis (GSEA) implied 1,691 significant GO terms (padj ≤ 0.05), among which 223 terms belong to cellular components, 1,226 to biological processes, and 242 to molecular functions.The KEGG-based GSEA significantly enriched 57 pathways (padj ≤ 0.05). Enriched GO and KEGG terms are listed in the supplementary data (see supplementary files 3, 4). The top 10 activated and suppressed GO terms were visualized in a dot-plot (Fig. 4), with dot size reflecting each term's gene counts. Within the top 10 activated GO terms, the majority of enriched genes belong to the ‘plasma membrane’ (GO:0,005,886), ‘extracellular region’ (GO:0,044,421), and ‘immune system process’ (GO:0,003,008). The most significant suppressed GO terms were ‘mRNA modification’ (GO:0,016,556) and ‘establishment of mitotic spindle orientation’ (GO:0,040,001). Interestingly, the GSEA-based GO analysis revealed a negative running enrichment NES = -2.3 score of 52 genes participating in the ‘Regulation of microtubule cytoskeleton organization’ (GO:0,070,507) (Fig. 5a, b) and a positive running enrichment score NES = 1.31 of 185 genes activating the ‘actin cytoskeleton organization’ (GO:0,030,036) (Fig. 5c, d). The down-expressed genes included *GNAI1* (G Protein Subunit Alpha I1), *PSRC1* (Proline And Serine Rich Coiled-Coil 1), *CEP131* (Centrosomal Protein 131), *RASSF7* (Ras Association Domain Family Member 7), and *CCNF* ( Cyclin F) (Fig. 5b). However, *ACTC1* (Actin Alpha Cardiac Muscle 1), *MYOC* (Myocilin), *BST2* (Bone Marrow Stromal Cell Antigen 2), *TACSTD2* (Tumor-Associated Calcium Signal Transducer 2), and *CSF1R* (Colony Stimulating Factor 1 Receptor) were among the top up-regulated genes (Fig. 5d). Likewise, the RhoGDI signaling pathway (Fig. S2) showed a significant downregulation (Inhibition) in hBMECs infected with A/WSN/33 (z-score = -3.2), which is known for its critical role in the organization of actin cytoskeleton. The pathway displayed an activated level of the F-actin. The ERM and Rho families, that function as cytoskeletal linkers and key regulators to the actin cytoskeleton, were proposed to downregulate. Consistent with our previous result (Fig. 2), we observed that during A/WSN/33 infection, a considerable number of genes that control the cytoskeleton and cell mobility were dysregulated in hBMECs.

### The innate immune and inflammatory response of hBMECs to influenza A virus

**Fig. 6:**
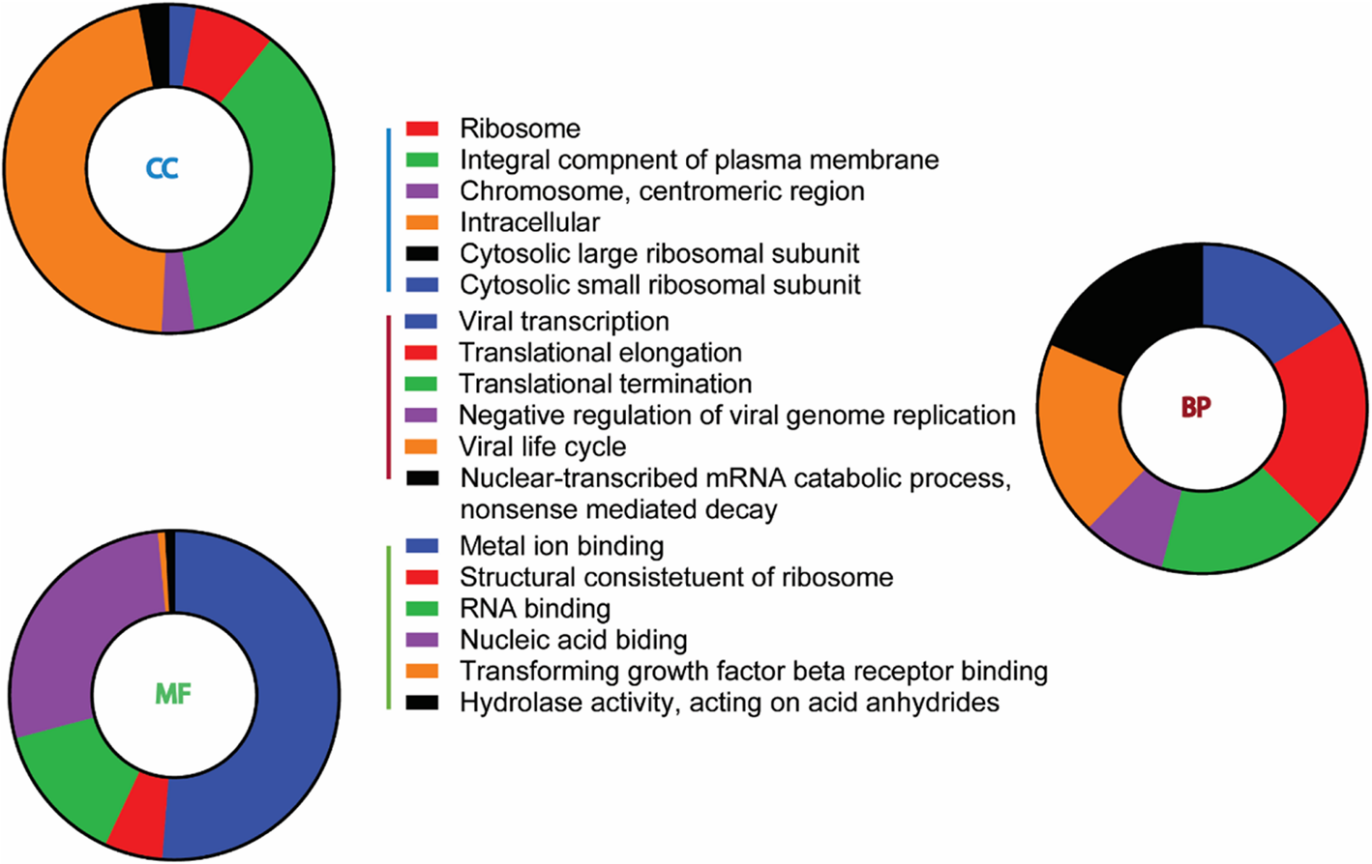
ORA gene ontology results from innate DB. Each pie chart represents the top six GO terms of the (CC) cellular components, (BP) biological processes, and (MF) molecular functions (padj ≤ 0.05)

For more details, the DEGs list was uploaded to InnateDB to obtain an over-representation analysis (ORA) for innate immune-related GO. The highest significant six GO terms of cellular components, biological processes, and molecular functions were observed in a pie chart according to their corresponding gene counts (Fig. 6). In addition, altered expression was found in the genes participating in the ribosome and the cytosolic ribosomal subunits of cellular components. Accordingly, the enrichment map generated a cluster of enriched biological processes with similar genes overlapped, yielding a dense interaction network between a set of GO terms. The network linked multiple GO terms, including plasma membrane, immune effector process and centered with the immune system process (Fig. S3a). Consequently, findings of the functional enrichment analysis of Fig. 4 and Supplementary Fig. 3a, in addition to the ridge-plot of Fig. S3b, illustrated that the immune and inflammatory responses were highly integrated into the biological changes associated with hBMECs infection with A/WSN/33 influenza virus at 12 hpi.

**Fig. 7:**
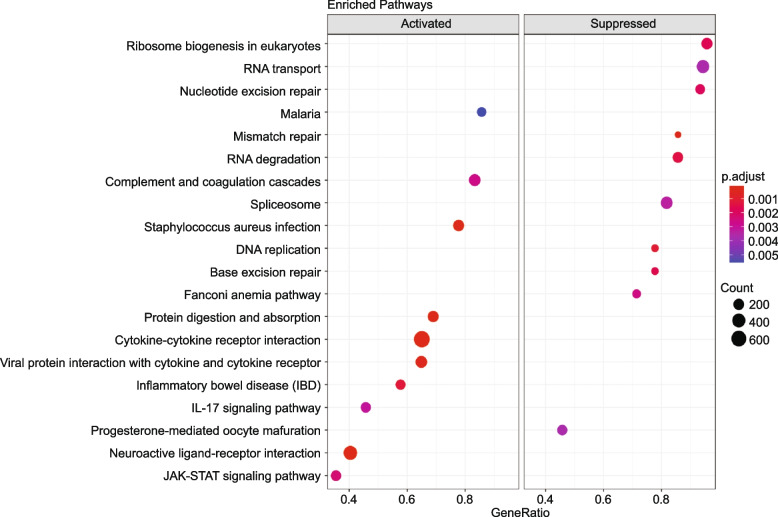
KEGG Pathway analysis. KEGG-based GSEA pathways are plotted in the order of gene ratios. The dots' size depicts the count number of the genes significantly differentiated in the KEGG pathway list, and the color represents the *p*-adjusted value (padj ≤ 0.05). GSEA, gene set enrichment analysis

**Fig. 8:**
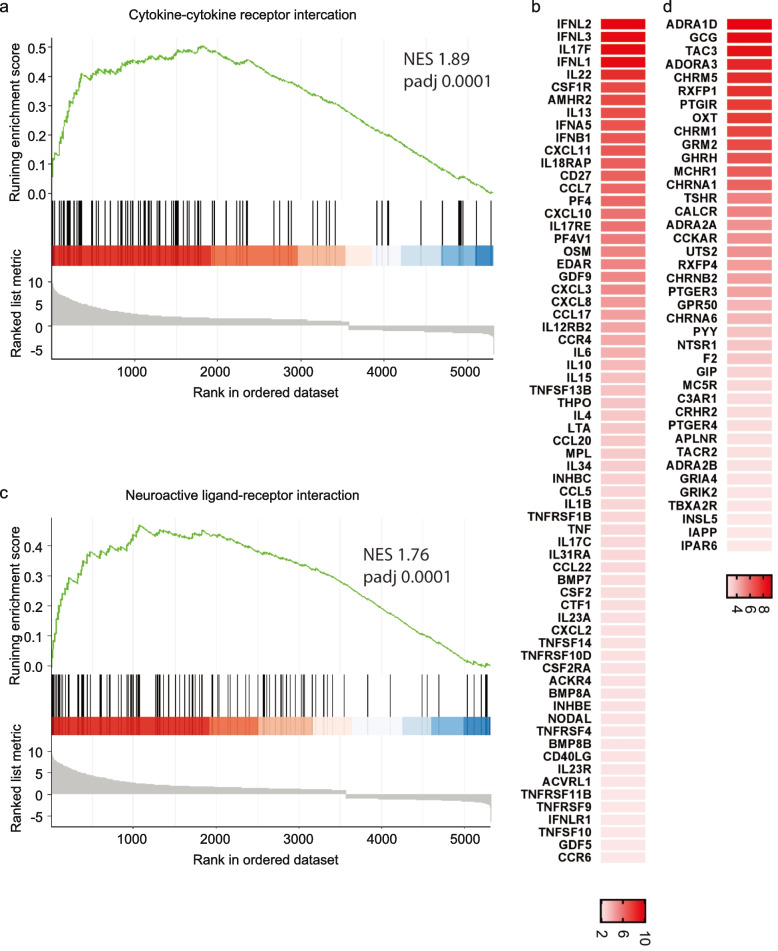
GSEA plots of enriched KEGG pathways. **a** GSEA plots indicating the running enrichment score for gene expression signature of ‘cytokine-cytokine receptor interaction’ (NES = 1.89, padj = 0.0001), and its associated top up-regaulted genes (**b**) visualized in a heatmap, in addition to the GSEA plot for the (**c**) ‘neuroactive ligand-receptor interaction’ pathway (NES = 1.76, padj = 0.0001) and its associated top activated genes (**d**). Black bars underneath the graph present the rank positions of genes from the gene set, the green line refers to the enrichment profile. GO, KEGG and GSEA were performed by the R package clusterProfiler; R package DOSE; R package org.HS.eg.db. and visualized by the R package Enrichplot and R package ggplot2

On the other hand, GSEA-based KEGG depicted the top significant 20 pathways, among which 10 were activated, while the others were suppressed (Fig. 7). Particularly, a high number of the up-regulated DEGs were predominantly enriched in the ‘cytokine-cytokine receptor interaction’ pathway (NES = 1.89, padj = 0.0001), (Fig. 8a, b) and ‘neuroactive ligand-receptor interaction’ pathway (NES = 1.7, padj = 0.0003), (Fig. 8c, d). The ridge-plot showed the expression distributions of core enriched genes for the enriched categories of GSEA, interpreting the pathways responding to the viral infection. The pathways were all activated associated with ‘viral protein interaction with cytokine and cytokine receptor’, ‘JAK-STAT signaling’, ‘RIG-I-like receptor signaling, and ‘NOD-like receptor signaling’ pathways were all activated. On the other hand, ‘DNA replication’ and ‘RNA degradation’ pathways were down-regulated (Fig. S4a). Likewise, the upset plot visualizeed the genes overlapping in different gene sets by plotting the fold change distributions with various categories (Fig. S4b).

**Fig. 9:**
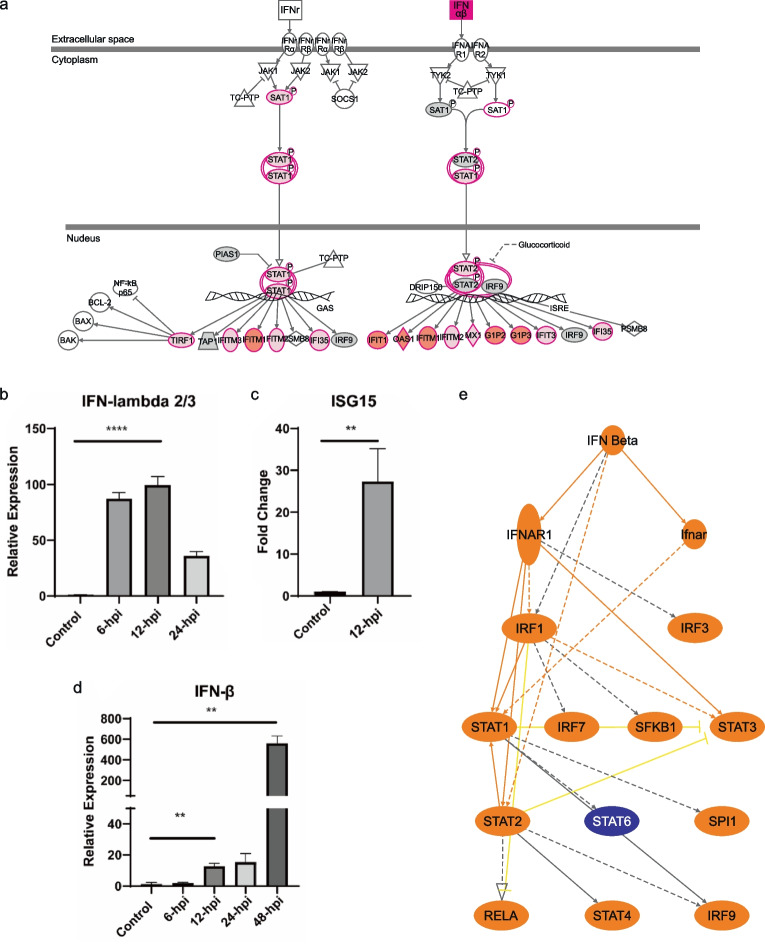
Regulation of induced immune genes expression in human brain microvascular endothelial cells (hBMECs) following influenza A virus infection. **a** Canonical pathway of the “Interferon signaling” for the genes differentially expressed in hBMECs 12 hpi, genes that are significantly up-regulated are shown in red. The intensity of red corresponds to an increase in fold change levels of the cells infected with A/WSN/33 (H1N1) compared to the control cells z-score = 4.2. White nodes specify genes with no significant gene expression at 12 hpi. The pathway was generated with IPA (Ingenuity pathways system). **b** quantification of gene expression by (RT-qPCR) for IFN-λ 2/3 (**c**) ISG15, and **d** IFN-β. (b-d) the hBMECs were infected with A/WSN/33 (H1N1) at MOI 0.1, and the target genes were quantified by relative quantification qPCR. statistical-significance analyzed by t-test * *p* < 0.05, ** *p* < 0.01, and **** *p* < 0.0001. **e** Mechanistic network by IPA for the upstream regulators interacting with IFN-β, which enables to discover plausible sets of connected upstream regulators that can work together to elicit the gene expression changes observed in our dataset

Viruses activate the innate immune system through the PRRs, including the Toll-like receptors, RIG-I-like receptors, NOD-like receptors, and C-type lectin receptors *(*Amarante-Mendes et al. 2018*)*. They also initiate the antiviral immune response by inducing the transcription of interferon and inflammatory molecules. IPA canonical pathways indicate the activation of PRRs, interferon signaling, TREM1 signaling, and neuroinflammation signaling pathway (Fig. S5a). The interferon signaling pathway (Fig. 9a, z-score = 4.2) indicated the induction and up-regulation genes among its downstream cascade, including *ISGs*, *IFITs*, and *IFIs*. To further support our results, we performed qPCR of the type III IFN-λ 2/3 (Fig. 9b) at different infection time points. The transcriptional activity of the mRNA significantly increased during the 6 to 12 hpi and dramatically decreased at 24 hpi. However, type I *IFNs* can actively induce *ISG15* up-regulation (Fig. 9c). *IFN-β* mRNA increased significantly from 12 hpi to reach its peak, recording a 500-fold change compared to the control hBMECs at 48 hpi (Fig. 9d).

Moreover, IPA upstream regulator analysis was conducted to identify the genes that might work as regulators for the underlined DEGs in our dataset and predicted whether they were activated or inhibited. *IFN-β* has been significantly up-regulated in our dataset and acted as a positive upstream regulator within the IPA analysis. The regulatory network of *IFN-β* (Fig. S5b, z-score = 5.6) predicted a downstream effect on activating the proinflammatory cytokines, including *TNF*, *IL-6*, and *IL-1β* and several types of *ISGs* while underlying the inhibition of *TGFBR1* and *CLK2*. Similarly, *INFL1* (Fig. S6, z-score = 5) is a significant upstream regulator of several cytokine genes. The mechanistic network (Fig. 9e) of *IFN-β* showed a plausible set of connected upstream regulators contributing to gene expression changes observed in our dataset. It predicted the activation of cell surface receptor *IFNAR* that has further activated *STAT1*, *STAT2*, and *STAT3* transcription factors. At the same time, the regulator effects demonstrated the methodology by which the activated upstream regulator *IFN-β* and its downstream effects might cause a potential inhibition of the ‘replication of viral replicon’ (Fig. S7). Briefly, the A/WSN/33 virus infection strongly induced the innate and inflammatory immune response of hBMECs.

### Alteration in the mitochondrial molecular function

**Fig. 10:**
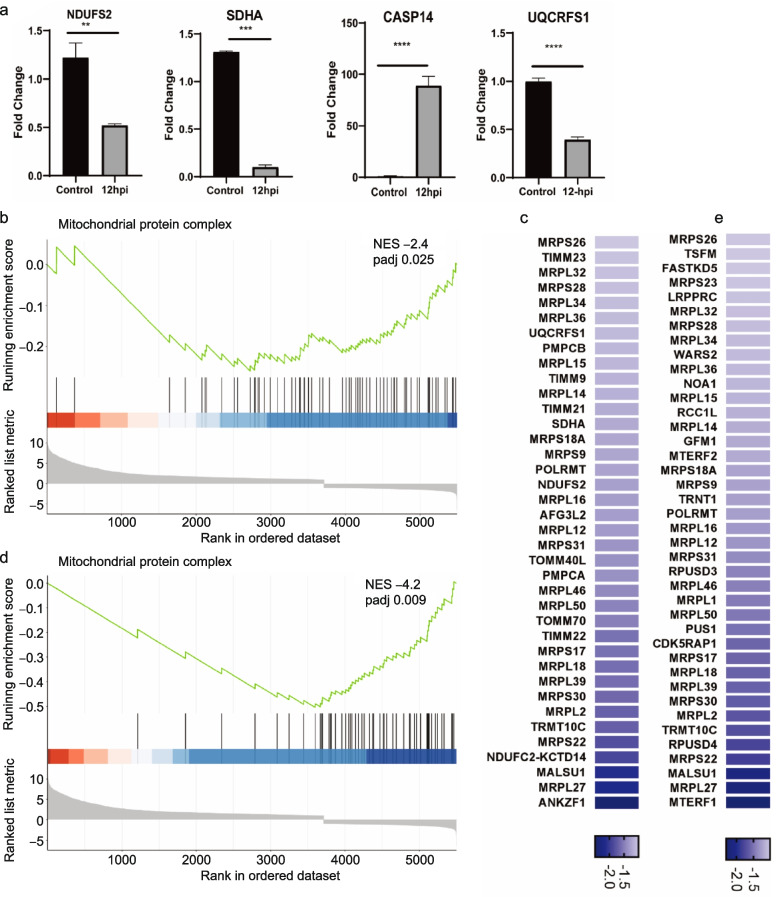
Altered mitochondrial gene expression of human brain microvascular endothelial cells (hBMECs). **a** quantification of gene expression by (RT-qPCR) showing the fold change increase or decrease for four mitochondrial genes expression *NDUSF2*, *SDHA*, *CASP14 *and *UQCRFS1* within the infected hBMECs cells at 12 hpi compared to the control cells. statistical-significance analyzed by t-test * *p* < 0.05, ** *p* < 0.01, *** *p* < 0.001, and **** *p* < 0.0001. **b** the GSEA running enrichment score for the suppression of ‘mitochondrial protein complex’ (NES = -2.4, padj = 0.025) enriched GO term and **c** its top downregulated genes. **d** indicates the GESA plot for negative running enrichment score of ‘mitochondrial gene expression’ (NES = -4.2, padj = 0.009) and **e** the top participating downregulated genes visualized in a heatmap. Black bars underneath the graph present the rank positions of genes from the gene set, the green line refers to the enrichment profile

We are now curious if the hBMECs mitochondrial system were affected by A/WSN/33. Several genes associated with mitochondrial functions have developed significant expression changes in the transcriptomic analysis, some of which have been confirmed by qPCR (Fig. 10a).

On the other hand, the *NDUFS2* (NADH: Ubiquinone Oxidoreductase Core Subunit S2), when mutated, causes dysfunction in the mitochondrial complex I, while the *UQCRFS1* (Ubiquinol-Cytochrome C Reductase, Rieske Iron-Sulfur Polypeptide 1) is inherently functioning in the mitochondrial complex III. The *SDHA* (Succinate Dehydrogenase Complex Flavoprotein Subunit A) gene encodes a major catalytic subunit of succinate-ubiquinone oxidoreductase. The three genes mentioned above are considered mandatory in the respiratory chain. And exhibiting a dramatic decrease in their expression might exacerbate the incidence of respiratory chain dysfunction, which is common with neurodegenerative diseases such as Alzheimer’s and Parkinson’s. Additionally, Caspase 14 (*CASP14*) induced following an apoptotic stimulus, along with *CASP10*, *CASP6*, and *CASP8* of DEGs, were all deregulated and implicated in principally participating in the programmed cell death. Withal, the running enrichment of the ‘mitochondrial protein complex’ (GO:0,098,798) and ‘mitochondrial gene expression’ (GO:0,140,053) were both negatively scored (NES = -2.4, padj = 0.02), (NES = -4.2, padj = 0.009) (Fig. 10b,c and d, e). The data above and the GSEA indicated that mitochondria could have played a role in hBMECs disruption during Influenza A virus infection.

### Neurodegeneration induced pathways in A/WSN/33 infected hBMECs

**Fig. 11:**
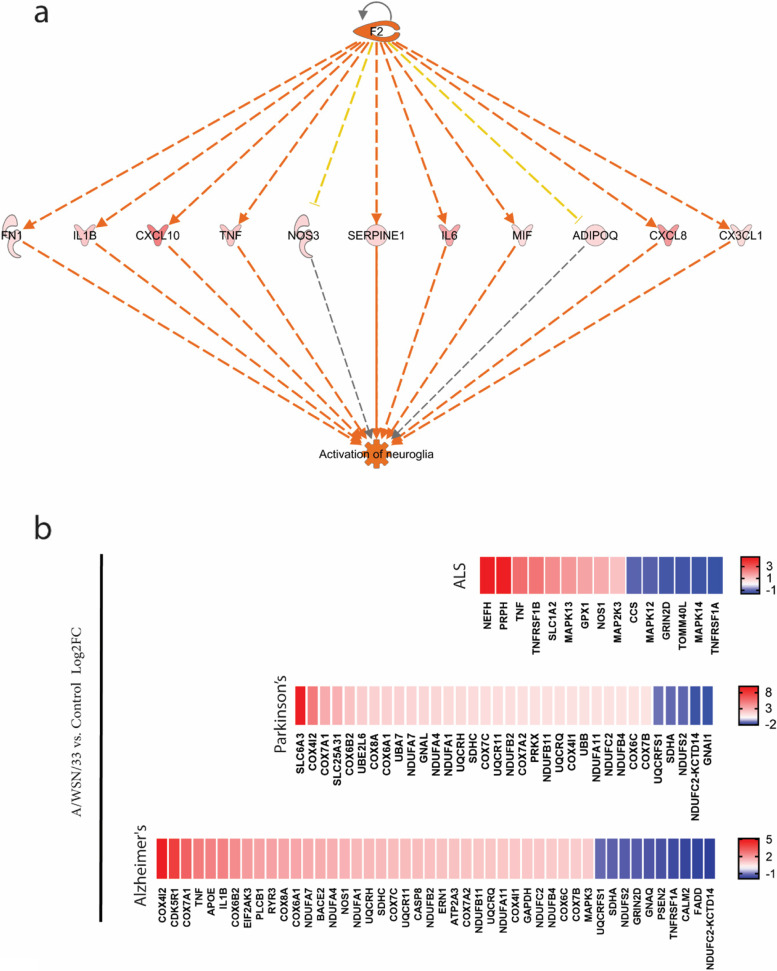
Enrichment analysis indicating neurological disorders. **a** The IPA regulator effects analysis shows that the F2 upstream regulator and its downstream effects indicate a potential activation for the neuroglia in hBMECs at 12 hpi. The regulator effects algorithm generates hypotheses that explain how the activation or inhibition of regulators leads to an increase or decrease of function. **b** the heatmaps reveal the genes participating in neurodegenerative disease pathways enriched and sorted according to Log2FC ratios in the DEGs list

The above results showed an inflammatory response triggered by virus infection to hBMECs, mainly by activating PRRs interaction, interferon signaling, and neuroinflammation signaling pathways, followed by the production of several interferon-stimulating genes (Fig. S5a). The IPA regulatory effect suggested that the inflammatory-induced *F2* gene might be involved in neuroglia activation, mainly through mediating its targets, including *FN1*, *IL1B*, *CXCL10*, *TNF*, *NOS3*, *SERPINE1*, *IL6*, *MIF*, *ADIPOQ*, *CXCL8* and *CX3CL1* (Fig. 11a). Since neuroinflammation is highly associated with neurodegenerative diseases, such as Alzheimer’s, Parkinson’s, and Amyotrophic lateral sclerosis (ALS), we determined their associated enriched disease pathways and the DEGs participating in each pathway (Fig. 11b). In the case of the genes significantly associated with Parkinson’s and Alzheimer's diseases, they were mainly a part of the mitochondria, including the cytochrome c oxidase genes (*cox*), *NDUF* subunits of NADH and *UQCRH* gene families, that all are associated with the respiratory electron transport of mitochondria. The uniquely enriched ALS genes were more associated with the Mitogen-Activated protein kinase *MAPK12*, *MAPK13* and *MAPK14* and the *TNF* and *TNF* receptor superfamily genes (*TNFRSF*). On the other side, the IPA network analysis specified a number of genes that play a role in central nervous system development, revealing a reduced gene expression for the brain-derived neurotrophic factor (BDNF) (Fig. S8). The results indicated that the Influenza A/WSN/33 virus could induce neuroinflammation and neurodegeneration pathways in hBMECs, which supports the hypothesis of previous literature that the influenza A virus induces symptoms like neurological diseases.

## Discussion

This study provides considerable insights into the potential response of the hBMECs to the pathogenesis of the Influenza A virus. The BBB disruption can be initiated by the cellular damage of brain endothelial cells, which could be followed by impaired brain homeostasis that induces an inflammatory immune response, neuronal cell death, and neurodegeneration (Sweeney et al. 2018; Erickson and Banks 2013; Parodi-Rullan et al. 2019; Rumbaugh and Nath 2009; Stolp et al. 2013). Some preliminary work was carried out to study the influenza virus's influence on causing brain diseases (Jurgens et al. 2012; Hosseini et al. 2018). However, we did not find enough answers about the impact of the influenza virus on the BBB and whether it causes inevitable damage to the hBMECs. We used a neurotropic strain of influenza virus A/WSN/33 (H1N1) to infect the hBMECs. The A/WSN/33 virus efficiently invaded the hBMECs, which was noticed on the morphological basis by induced cell damage and molecular basis by the confirmed viral replication through RT-qPCR and protein expression through western blotting. Accordingly, the RNAseq at 12 hpi revealed a set of DEGs with an increased level of immune-related and antiviral genes, including type I and III interferons, interferon-stimulated genes, proinflammatory cytokines, and chemokines such as *IFN-β*, *IFN-λ*, *TNF*, *IFI44*, *ISG15*, *IL-6*, *CXCL2*, *CXCL3*, *CXCL8*, *CXCL10*, *CXCL11* and *CXCL16*. These results are consistent with previous research studying the influenza virus interaction with mouse cortical neurons (Wang et al. 2016).

The influenza virus NP translocates inside the cells by existing in the nucleus during early infection; later, it spreads into the cytoplasm and binds to the F-actin through specific residues (Neumann et al. 1997; Zheng and Tao 2013; Digard et al. 1999). Using immunofluorescence, we noticed an accumulation of the virus NP in the hBMECs nucleus that was scattered in the cytoplasm to form a colocalization with the actin cytoskeleton. Previous studies have displayed that the actin network plays a major role in virus replication (Kumakura et al. 2015; Gupta et al. 1998). The virus tends to be reduced by inhibiting the actin-myosin formation (Kumakura et al. 2015). Additionally, a research study has indicated that cellular actin is necessary to activate human parainfluenza virus type 3 (HPIV3) transcription (Gupta et al. 1998). The infected hBMECs cells showed a morphological change in the actin cytoskeleton structure compared to intact cells (Fig. 2), coinciding with the activation of the gene set responsible for actin regulation (Fig. 5a-d). The genes, activation could play a central role in intracellular transport, particle movement at the cell periphery before virus fusion, and the NP transcription, replication, and trafficking, such as *ACTC1* gene (König et al. 2010). Thus, we speculate that the influenza virus might enhance the cytoskeleton re-organization by increasing the F-actin ratio (Lakadamyali et al. 2003). A similar conclusion revealed that the virus transport mechanism following endocytosis was mainly actin-dependent. That was further replaced by the microtubule-based movement to the nucleus site where the viral RNA synthesis occurs, while the NP and F-actin interaction is followed by cytoplasmic retention (Lakadamyali et al. 2003; Digard et al. 1999). Consistently, the cytoplasmic retention by NP might illustrate the reason behind the doughnut shape or the condensation of the F-actin towards the cell edges. The number of activated genes participating in the actin cytoskeleton organization was higher (185) than suppressed in the microtubule cytoskeleton organization (52) (Fig. 5a,c). Interestingly, the microtubules are known to mediate host responses to infection; they facilitate the antiretroviral activity of the TRIMs family (Elis et al. 2015). Viruses can still evolve countermeasures to replicate successfully. For example, rabies virus P protein switches the host antiviral *STAT1* from MT-facilitated to an MT-inhibited nuclear import process. Besides the virus’s ability to block antiviral host responses, some viruses can also cause cellular-induced dysregulated cell division (Naghavi and Walsh 2017). Although most of the genes functioning in the cytoskeleton organization were up-regulated following the IAV infection, a considerable number of genes were also suppressed. The exact roles played by the influenza virus on the microtubule organization are still under study; however, we predict from previous literature that the virus could induce the genes’ suppression to fight against the antiviral host response.

The virus entrance can be detected by the activation of PRRs that include the activation of Toll-like receptors, RIG-I-like, and NOD-like receptors (Amarante-Mendes et al. 2018; Takeuchi and Akira 2010; Chen et al. 2013). The antiviral genes are induced by the activation of the type I interferon signaling, which builds the first line of the host defense against the virus infection (Schneider et al. 2014; McNab et al. 2015). The influenza virus likely induces the activation of PPRs in the hBMECs and activates the interferon signaling pathways to initiate a cascade of interferon-stimulated genes. Likewise, our results demonstrate a significant upregulation in the genes playing a crucial role in the cytokine-cytokine receptor interaction, JAK-STAT, neuroinflammation and neuroactive ligand-receptor pathways. With slight differences from other findings discussing astrocytes' response to Influenza A virus (Lin et al. 2015), hBMECs are likely more susceptible to the virus infection than astrocytes. The neuroactive ligand-receptor interaction pathway was activated earlier in hBMECs at 12 hpi than astrocytes at 24 hpi. In addition, downstream cascade of IFN signaling pathway was also less induced in astrocytes at 24 hpi than in hBMECs at 12 hpi (Lin et al. 2015).

Rho GTPases are core regulatory molecules that link the surface receptors to organize actin and microtubule cytoskeleton (Bozza et al. 2015). On the other hand, we investigated that during the IAV infection of hBMECs, there was an evident decline in the genes functioning in the RhoGDI signaling pathways. Thus, the ERM and Rho families cytoskeletal regulators showed an increased inhibition, while the F-actin was activated.

According to previous studies, mitochondrial dysfunction is considered a primary reason for several neurological disorders (Wu et al. 2019). Apoptosis is the principal pathological feature of neurodegeneration, which is controlled by the mitochondria (Wu et al. 2019; Hroudová et al. 2014). Our findings showed that the hBMECs infected with the influenza virus revealed several defects within the mitochondria, showing a pattern of inhibition within most of their biological processes, specifically in the electron transport chain, which plays a significant role in the pathogenesis of AD.

Few studies have examined the longer-term neurologic consequences due to influenza virus infection. Jang et al*.* tracked the H5N1 virus once introduced to the mouse; they reported that the virus traveled from the peripheral nervous system into the CNS to higher neuroaxis levels (Jang et al. 2009). They also indicated a significant loss of the dopaminergic neurons by 17% in the SNpc 60 days following H5N1 infection (Jang et al. 2009). Although the virus had disappeared from the brain in 21 days, there was long-lasting microglial activation (Jang et al. 2009). Moreover, a group of researchers specified more information about the long-term consequences of CNS infection with neurotropic and non-neurotropic IAV strains (Hosseini et al. 2018). They outlined a significant spine loss in the hippocampus and a microglia activation during the acute phase of the disease with the neurotropic H7N7 and the non-neurotropic H3N2 in mice (Hosseini et al. 2018). Both neurotropic and non-neurotropic strains indicated a significant reduction in spine number 30 days post-infection. Complete recovery was noticed at 120 dpi, providing evidence for long-term disturbances in the CNS (Hosseini et al. 2018).

By casting a light on the previous research, we conclude that s neurotropic viruses such as the human immunodeficiency virus (HIV) and the Japanese encephalitis virus could invade the CNS and cause diseases. We are currently more curious about how some viruses could spread from the respiratory system to the CNS and induce diseases. It is not only the influenza viruses but also the syncytial virus (hRSV), the human metapneumovirus (hMPV), and coronavirus (CoV) that have been all detected in the cerebrospinal fluid (CFS) following infection (Bohmwald et al. 2018). The hRSV, which induces a respiratory illness in infants, also alters the neurologic homeostasis with seizures, ataxia, and other encephalopathy symptoms (Espinoza et al. 2013). It translocates from the lung to invade the CNS through a hematogenous/blood–brain barrier route and releases humoral neurotoxic cytokine mediators (Park and Suh 2014). The hMPV respiratory pathogen severely infects newborns' respiratory systems and immunocompromised individuals (Shafagati and Williams 2018; Edwards et al. 2013). During the last two decades, hMPV have been associated with a potential neuroinvasion. They reported different cases of febrile seizures, encephalitis, and encephalopathies parallel to the existence of the virus RNA in the CSF (Jeannet et al. 2017; Sánchez Fernández et al. 2012; Desforges et al. 2019). In a similar pattern to the influenza virus, the HCoV could enter the CNS through the olfactory bulb upon nasal infection (Arbour et al. 2000). Neuroinvasion was noticed along with the presence of the HCoV RNA in brains, as it was detected in the human brain parenchyma of patients with multiple sclerosis (Arbour et al. 2000; Burks et al. 1980). Likewise, the primary glial, when infected with HCoV secretes TNF-α, IL-12p40, IL-15, IL-6, CXCL9 and CXCL10 (Bohmwald et al. 2018). The recent coronavirus, SARS-CoV-2, utilizes the angiotensin-converting enzyme 2 (ACE2) receptor to enter the cells (Baig et al. 2020). It can attack the CNS by targeting the ACE2 receptors expressed on the neurons, glial tissues, and brain vasculature, as it was further associated with neurological manifestations (Turner et al. 2004; Kabbani and Olds 2020; Ahmed et al. 2020).

To conclude, neurodegenerative disorders such as AD, Parkinson’s and ALS, usually observed in elderly persons, are characterized by neuronal cell death. They could be activated following virus attacks by several inflammatory processes (Chen et al. 2016). As long as neuronal cell death is not regenerated, we speculate that successive infection with the influenza virus might play a potential role in the appearance of neuronal disorders over time. It is also essential to realize that understanding the underlying interaction between neuronal and inflammatory immune cells could solve a future problems for the various neurodegenerative disorders. For example, senile plaques and neurofibrillary tangles of AD could induce a neuroinflammation route that triggers the pathogenesis of the disease as much as or even more than the plaques themselves (Zhang et al. 2013; Heneka et al. 2015). AD pathogenesis progression occurs after a robust immunological interaction. The misfolded protein binds to the PRRs on astroglia and microglia and further induces innate immune and proinflammatory responses (Heneka et al. 2015). The incidence of symptoms like neurodegenerative disease or the activation of their related pathways following infection with the Influenza A virus should be critical for answering the upcoming research questions regarding the induction of AD and other neurodegenerative disorders in the long run. Moreover, it might also be a useful study model for future therapeutics production and neurodegenerative disease prevention.

## Conclusions

Our results demonstrate the susceptibility of hBMECs as a part of the BBB to become infected by A/WSN/33. The infection is followed by robust innate immune and inflammatory signaling activation, disruption in the cell morphology and cytoskeletal structure, dysfunction on the mitochondrial level, and activation of neuroglia and neurodegenerative disease pathways.

## Materials and methods

### Cells and viruses

The hBMECs were given generously by Dr. Xiangru Wang (Huazhong Agricultural University) and initially obtained from Prof. Kwang Sik Kim at Johns Hopkins University School of Medicine (Yang et al. 2016; Stins, Badger, and Sik Kim 2001; Stins et al. 1997). The cells were cultured in a T25 flask containing Dulbecco’s modified Eagle’s medium (DMEM) supplemented with 10% FBS (Gibco), 2 mM l-glutamine, 1% MEM non-essential amino acid solution, 1 mM sodium pyruvate, 1% MEM amino acid solution, 1% MEM vitamin solution and 100 U/mL penicillin/streptomycin. The cells were incubated at 37°C under 5% CO_2_ until the monolayer reached confluency. Madin-Darby Canine Kidney (MDCK) cells were used for virus titration. The cells were cultured in DMEM (Invitrogen) supplemented with 100 U/mL penicillin/streptomycin and 10% FBS (Gibco) at 37°C under a 5% CO_2_ incubator. A/WSN/33 (H1N1) virus strain offered by Prof. Hongbo Zhou (Huazhong Agricultural University) was propagated using 10-day-old embryonic chicken eggs, titrated, and preserved at -80°C.

### hBMECs infection with A/WSN/33 (H1N1)

hBMECs were cultured in a growth medium in a 12-well plate at 37°C, 5% CO_2,_ and further incubated for 24 h. We infected the cells with 7 × 10^6^ PFU/mL (0.1 MOI) of A/WSN/33 (H1N1). After two hours of virus infection, the DMEM supernatant with the unbound virus was discarded, followed by three washes of phosphate-buffered saline (PBS). Fresh DMEM with 2% FBS was added to the cells and incubated at 37°C under 5% CO_2_. Cells were collected at different time points for RNA and protein extractions.

### RNA extraction and cDNA library construction

Total RNA from the infected hBMECs with A/WSN/33 (H1N1) was collected using TRIzol (Invitrogen, NY) following the manufacturer’s procedure. RNA concentration was confirmed by NanoDrop to ensure RNA quality before cDNA library construction. One µg of the total RNA was used for the BGISEQ-500 library construction, and double-stranded DNA contaminants in RNA samples were degraded by DNase I. mRNA molecules were purified from total RNA by Oligo (dT)-attached magnetic beads and fragmented into small pieces. N6 random primers were used for dscDNA synthesis by reverse transcription, dscDNA were subjected to end repair and, 3′ end adenylated. Adaptors were ligated at the 3′ end, and PCR amplification was done using specific primers. Furthermore, the PCR product was denatured into single-stranded DNA and cyclized with splint oligo and DNA ligase to process the final library. The DNB (DNA nano ball) was then prepared and sequenced for SE50 (Fig. S1).

### RNA sequencing and annotation

The sequencing was performed by BGI-China following the BGISEQ-500 platform, generating 23,761,511 kb of clean reads after the removal of low-quality reads. The data were confirmed for clean reads by FastQC for quality control, then mapped and assembled to the human reference genome GRCh38 (hg38) following the HISAT2/StringTie protocol (Kim et al. 2015). The annotation and differentially expressed genes were obtained using the DESeq2 R package with considerable significance at a 5% (0.05) *p*-adjusted value, and “apeglm” tool was used for log fold change shrinkage (Love 2014; Zhu et al. 2019). Differentially expressed genes were observed in a volcano plot using the R package Enhanced volcano (Blighe K 2020). Gene ontology GO, KEGG, and GSEA resulted from the R package clusterProfiler and the R package DOSE (Yu et al. 2012, 2015). The figures were visualized using the R package enrichplot and ggplot2 (Yu 2019; Wickham 2009). The differentially expressed genes were also uploaded to the database of InnateDB to enrich the innate immune-related functions (Breuer et al. 2013). The canonical pathway, upstream regulators, and network analysis were generated through IPA (Ingenuity Pathway Analysis, QIAGEN Inc.).

### Western blotting

After 12 h of infection, hBMECs were washed twice with ice-cold PBS and collected using a radioimmunoprecipitation assay (RIPA) containing protease inhibitor cocktail (Roche) and phosphatase inhibitor cocktail (Roche). The cells were homogenized using a sonicator machine (Qsonica LCC, USA), followed by centrifugation at 10,000 *g* for 10 min at 4°C. The cell debris was discarded, and the protein concentration was measured with a BCA protein assay kit (Beyotime, China). Moreover, the protein was electrophoretically separated on a 10% sodium dodecyl sulfate–polyacrylamide gel electrophoresis (SDS-PAGE). We then transferred the proteins to polyvinylidene difluoride (PVDF) membranes 0.22 µm (Bio-Rad, CA). The membrane was soaked in 5% non-fat-containing milk in Tris-buffered saline with 0.1% Tween 20 and blocked for two hours at room temperature. It was then incubated overnight with the primary polyclonal anti-rabbit NP protein (GeneTex Inc., CA, USA), and rabbit anti-β-actin primary antibodies (Proteintech, China) at 4°C (as a loading control). After washing with TBST, the membranes were incubated with a goat anti-rabbit secondary antibody for one h at room temperature. Finally, all the signals were visualized using Chemiluminescent chromogenic substrate ECL.

### Real-Time Quantitative RT-PCR (qRT-PCR)

Total RNA of hBMECs was isolated using TRIzol (Invitrogen, Grand Island, NY, USA).The RNA was reverse-transcribed into cDNA by 5X All-In-One RT MasterMix (abm, Canada). The qPCR was performed using RealUniversal Color PreMix SYBR Green (Tiangen, China) in ABI ViiA7 PCR system (Applied Biosystems, Foster City, CA, USA). qPCR was performed to evaluate the transcriptional levels of host response genes based on RNAseq data analysis. Expression was normalized to the β-actin reference gene levels, while relative expression was calculated using the comparative method of 2-∆∆Ct. The virus replication curve was quantified and normalized using virus copy numbers (Rüdiger et al. 2019; Frensing et al. 2016). Changes in gene expression were examined by t-test, and *p* < 0.05 was considered significant. All primers manipulated in this study are listed in (Table S1).

### Immunofluorescence and cell morphology

The cell morphology was observed at different time points after infection using Ph Microscopy by Nikon inverted microscope Ti-U (ECLIPSE, Japan). hBMECs were seeded at a density of 1.5 × 10^4^ per well on chamber slides and deposited at the bottom of a 24-well plate. The cells were inoculated with A/WSN/33 (H1N1) at 0.1 MOI and incubated at 37°C at 5% CO_2_ for 12 h. The slides were rinsed three times with 1 × PBS and further fixed with 4% paraformaldehyde (PFA) for 10 min at 37°C. After washing with PBS, the cells were permeabilized in 0.1% Triton X-100 in 1 × PBS at room temperature for 10 min, then washed again with PBS. Permeabilized cells were then blocked with 2% BSA in PBS for 1 h at room temperature and washed. For immunofluorescence, the cells were incubated with the primary anti-nucleoprotein NP (1:500; rabbit polyclonal, GeneTex Inc., CA, USA) diluted in 0.1% BSA and incubated for three hours at room temperature. The secondary antibody used was Alexa flour 647 goat anti-rabbit IgG (1:500; Thermofisher). After washing, the cells were stained for F-actin with fluorescent FITC-conjugated Phalloidin for 2 hat 37 °C and with Hoechst for 5 min. The slides were observed under a laser confocal microscope (Leica, Germany).

### Statistical analysis

The experiments were performed in triplicate and repeated three times. The values were shown as the mean ± standard error of the mean (SEM), and the statistical significance of the differences between groups was determined by Tukey’s post hoc tests and student’s* t*-test *p* < 0.05. The statistical analysis of differential gene expression profiles was done using R software (V. 3.6.3). The HISAT2 alignment tool was used for mapping RNA sequencing reads to the reference genome. StringTie assembler tool of RNAseq alignments was used, which uses a novel network flow algorithm as well as an optional *de novo* step of assembly. The StringTie’s output was processed by the DESeq2 R package to obtain the differentially expressed genes. Genes were listed with considering a level of significance less than 0.05 of adjusted *p-*value and a log_2_ fold change (Log2FC) of ± 1. The volcano plot was observed using the enhanced volcano plot R package with a cutoff for Log2FC at ± 2 and 0.05 of an adjusted *p*-value. The R package ClusterProfiler was applied using the GSEA and overrepresentation analysis for the hypergeometric test. The enrichment analysis was applied to the differentially expressed genes obtained from the DESeq2 analysis to determine the genes' functional distribution to GO terms and KEGG pathways using a cutoff for the *p*-adjusted value at 0.05. The figures were visualized with two R packages, enrichplot, and ggplot2. The canonical pathway, upstream regulators, and network analysis were generated through IPA (QIAGEN Inc.). IPA conducts core analysis using two statistical outputs. First, the *p*-value derived from a Right-Tailed Fisher’s Exact test to reflect the estimated association or overlapping between a set of significant molecules and a pathway or function that might be a result of random chance (The smaller the *p*-value, the likely the random association exists), which finally does not consider the directional effect. The second output is the z-score, or standard score, which is the number of standard deviations a given data point lies above or below the mean (Kramer, Green, and Pollard Jr 2014). A positive z-score indicates that the raw score is higher than the mean average (increases); a negative z-score reveals that the raw score is below the mean average (decreases). Graphs are plotted and analyzed by GraphPad Prism software version 8 (GraphPad, La Jolla, CA, USA). All figures were conducted in Adobe Illustrator (Adobe, Mountain View, CA, USA).

## Supplementary Information

**Additional file 1:** **Fig. S1.** cDNA library construction for RNA sequencing. mRNA enrichment with Oligo (dT) magnetic beads to select mRNA with poly-A tail, target RNA was fragmented and N6 random primers were used for reverse transcription producing a double-stranded cDNA (dscDNA). dscDNA fragments were end-repaired and 3’ adenylated, then the “T” of the adaptor was ligated with “A” at the 3’ end, two specific primers were designed to PCR amplify the ligation product. The PCR product was the denatured by heat and the single-strand DNA was cyclized by splint oligo and DNA ligase to format the final library, DNB was prepared and sequenced for SE50. **Fig. S2.** IPA canonical pathway of RhoGDI signaling. The IPA results indicate an overall inhibition of RhoGDI signaling activity during A/WSN/33 infection to hBMECs. The intensity of the red color indicates activation, while the green color's intensity indicates inhibition. **Fig. S3.** GO functional enrichment of hBMECs at 12 hpi. a Enriched GO terms of hBMECs organized in a network map with edges connecting overlapping gene sets. b Ridgeplot visualizing the expression distributions of core enriched genes for the top 30 significant enriched GO terms of hBMECs following A/WSN/33 (H1N1) infection, it also interprets the up and regulated GO terms. **Fig. S4.** KEGG functional enrichment of hBMECs 12 hpi a Ridgeplot KEGG based, visualizing the expression distributions of core enriched genes for GSEA enriched KEGG pathways of hBMECs following the virus infection. The plot determines the up and down-regulated pathways. b Upset plot visualizing the overlapped genes among different gene sets. **Fig. S5.** IPA Pathways and network analysis. a top 20 significant canonical pathways enriched based on the DEGs list uploaded to the IPA for hBMECs 12 hpi. “Role of pattern recognition receptors in recognition of bacteria and viruses” and “Interferon signaling” are the top two pathways enriched. The threshold indicates a minimum significance level –log (p-value) from Fisher’s exact test. The ratio refers to the number of molecules from the dataset that map to the pathway listed divided by the total number of molecules that define the canonical pathway from within the IPA knowledgebase. b The network explains the interaction between IFN-β as an upstream regulator and its target genes. **Fig. 6S.** Regulatory network explaining the interaction between IFNL1 as an upstream regulator and its target genes. **Fig. S7.** IPA regulator effects of IFN-β. Integrated results from IFN-β upstream regulator and its downstream effects indicate a potential inhibition for the “virus replication” in hBMECs following 12 h of A/WSN/33 infection. The regulator effects algorithm generates hypotheses that explain how the activation or inhibition of regulators leads to an increase or decrease of function. **Fig. S8.** IPA network analysis. The network reveals the molecules interacting together and functioning in the central nervous system development, Red indicates upregulation; green indicates downregulation. **Table S1.** List of primers used during the research study. This table shows the primers used for the RNAseq results validation by qPCR.

**Additional file 2.** DEGs list of hBMECs 12 hpi infection with A/WSN/33 (H1N1), the list is an output of the R package DESeq2 (|log2FC|≥1; padj<0.05).

**Additional file 3.** GO list of hBMECs 12 hpi with A/WSB/33 (H1N1), the list in an output of the R package clusterProfiler.

**Additional file 4.** KEGG list of hBMECs 12 hpi infection with A/WSN/33 (H1N1), the list is an output of the R package clusterProfiler.

## Data Availability

Data deposition: The sequence reported to this article for the hBMECs experiment has been deposited to the NCBI Sequence Read Archive (SRA) under the accession no. ( PRJNA615331).

## Abbreviations

BBB: Blood–Brain Barrier
CNS: Central nervous system
hBMECs: Human brain microvascular endothelial cells
DEGs: Differentially expressed genes
GO: Gene ontology
KEGG: Kyoto Encyclopedia of Genes and Genomes
IAV: Influenza A virus
BDNF: Brain-derived neurotrophic factor
NGF: Nerve growth factor
SNpc: Substantia nigra pars compacta
MOI: Multiplicity of infection
PCR: Polymerase chain reaction
ORA: Over Representation Analysis
GSEA: Gene set enrichment analysis
IFITs: An interferon-induced protein with tetratricopeptide repeats
ISGs: Interferon stimulating genes
IFIs: Interferon-induced proteins
IPA: Ingenuity Pathway Analysis
PRRs: Pattern recognition receptors
FBS: Fetal bovine serum
MDCK: Madin-Darby Canine Kidney
PBS: Phosphate-buffered saline
DNB: DNA nano ball
RIPA: Radioimmunoprecipitation assay
CSF: Cerebrospinal fluid
