# Supplementary material for: Altered gene expression in human brain microvascular endothelial cells in response to the infection of influenza H1N1 virus
Source: Anim Dis. 2022 Nov 3;2(1):25. doi: 10.1186/s44149-022-00053-9 (PMC9631584; doi:10.1186/s44149-022-00053-9)
Supplement: Supplementary file 1 — Additional file 1: Fig. S1. cDNA library construction for RNA sequencing. mRNA enrichment with Oligo (dT) magnetic beads to select mRNA with poly-A tail, target RNA was fragmented and N6 random primers were used for reverse transcription producing a double-stranded cDNA (dscDNA). dscDNA fragments were end-repaired and 3’ adenylated, then the “T” of the adaptor was ligated with “A” at the 3’ end, two specific primers were designed to PCR amplify the ligation product. The PCR product was the denatured by heat and the single-strand DNA was cyclized by splint oligo and DNA ligase to format the final library, DNB was prepared and sequenced for SE50. Fig. S2. IPA canonical pathway of RhoGDI signaling. The IPA results indicate an overall inhibition of RhoGDI signaling activity during A/WSN/33 infection to hBMECs. The intensity of the red color indicates activation, while the green color's intensity indicates inhibition. Fig. S3. GO functional enrichment of hBMECs at 12 hpi. a Enriched GO terms of hBMECs organized in a network map with edges connecting overlapping gene sets. b Ridgeplot visualizing the expression distributions of core enriched genes for the top 30 significant enriched GO terms of hBMECs following A/WSN/33 (H1N1) infection, it also interprets the up and regulated GO terms. Fig. S4. KEGG functional enrichment of hBMECs 12 hpi a Ridgeplot KEGG based, visualizing the expression distributions of core enriched genes for GSEA enriched KEGG pathways of hBMECs following the virus infection. The plot determines the up and down-regulated pathways. b Upset plot visualizing the overlapped genes among different gene sets. Fig. S5. IPA Pathways and network analysis. a top 20 significant canonical pathways enriched based on the DEGs list uploaded to the IPA for hBMECs 12 hpi. “Role of pattern recognition receptors in recognition of bacteria and viruses” and “Interferon signaling” are the top two pathways enriched. The threshold indicates a minimum significance level –log ( [file 44149_2022_53_MOESM1_ESM.pdf]

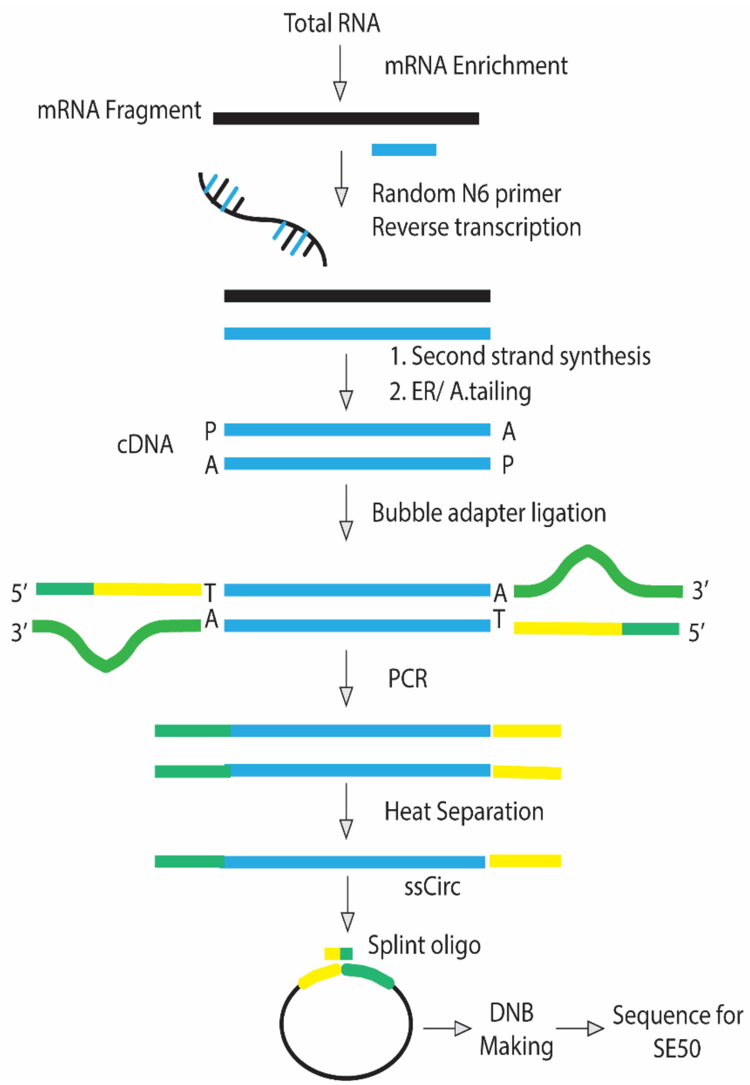

Fig. S1 cDNA library construction for RNA sequencing. mRNA enrichment with Oligo (dT) magnetic beads to select mRNA with poly-A tail, target RNA was fragmented and N6 random primers were used for reverse transcription producing a double-stranded cDNA (dscDNA). dscDNA fragments were end-repaired and 3’ adenylated, then the “T” of the adaptor was ligated with “A” at the 3’ end, two specific primers were designed to PCR amplify the ligation product. The PCR product was the denatured by heat and the single-strand DNA was cyclized by splint oligo and DNA ligase to format the final library, DNB was prepared and sequenced for SE50.

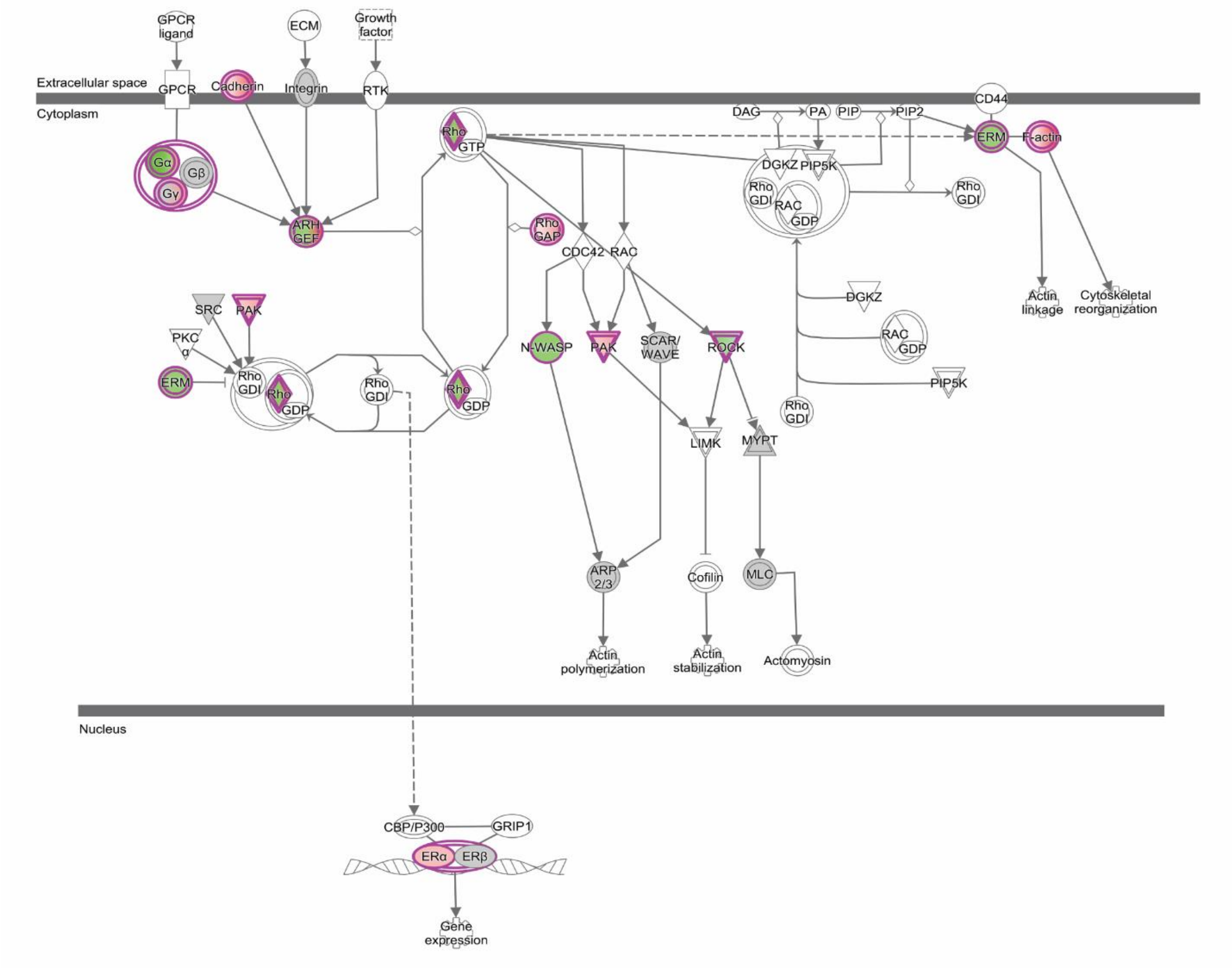

Fig. S2 IPA canonical pathway of RhoGDI signaling. The IPA results indicate an overall inhibition of RhoGDI signaling activity during WSN infection to hBMECs. The intensity of the red color indicates activation, while the green color's intensity indicates inhibition.

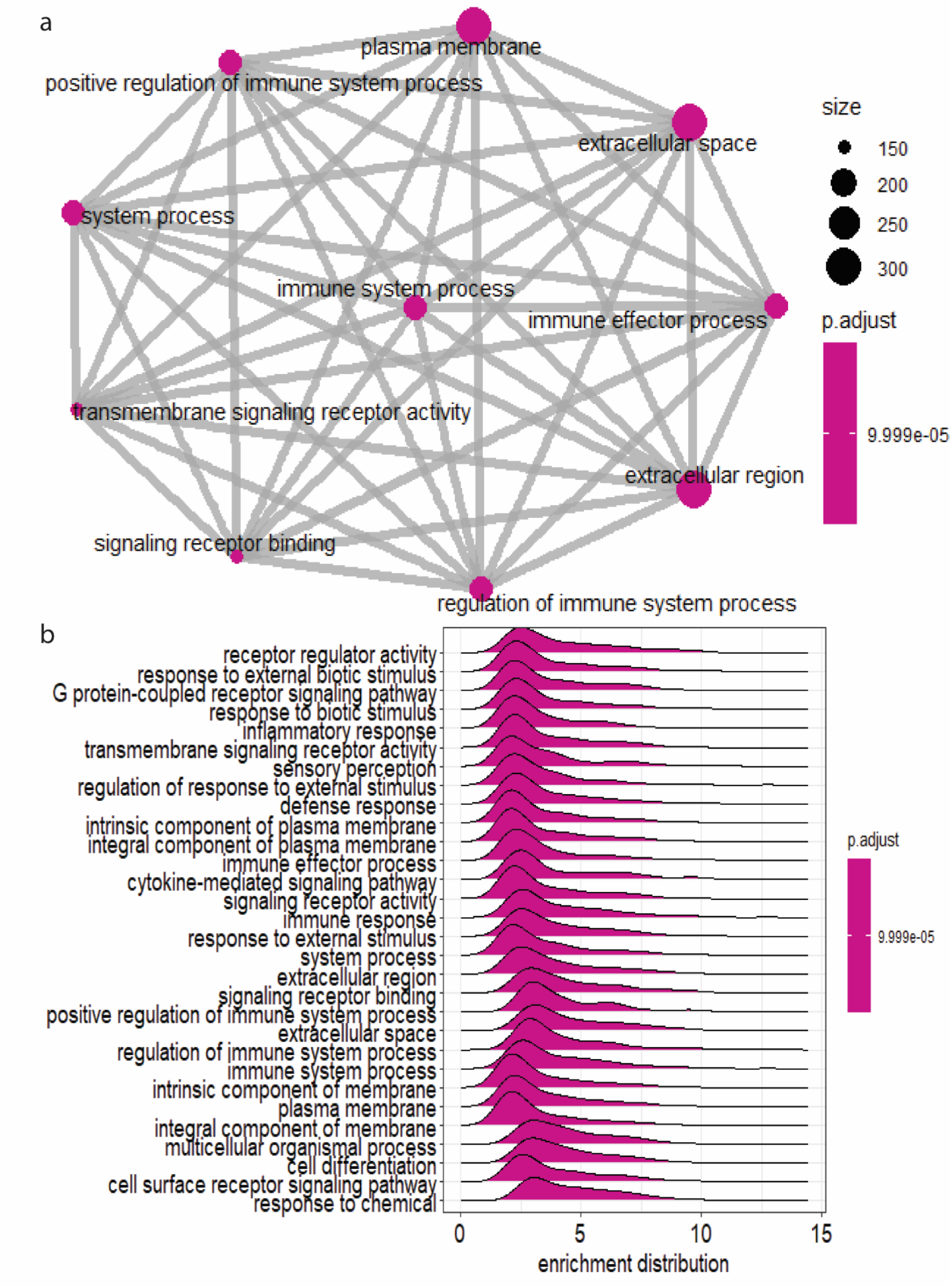

a
plasma membrane
positive regulation of immune system process
extracellular space
system process
immune system process
immune effector process
transmembrane signaling receptor activity
extracellular region
signaling receptor binding
regulation of immune system process
size
150
200
250
300
p.adjust
9.999e-05
b
receptor regulator activity
response to external biotic stimulus
G protein-coupled receptor signaling pathway
response to biotic stimulus
inflammatory response
transmembrane signaling receptor activity
sensory perception
regulation of response to external stimulus
defense response
intrinsic component of plasma membrane
integral component of plasma membrane
immune effector process
cytokine-mediated signaling pathway
signaling receptor activity
immune response
response to external stimulus
system process
extracellular region
signaling receptor binding
positive regulation of immune system process
extracellular space
regulation of immune system process
immune system process
intrinsic component of membrane
plasma membrane
integral component of membrane
multicellular organismal process
cell differentiation
cell surface receptor signaling pathway
response to chemical
0
5
10
15
enrichment distribution
p.adjust
9.999e-05

Fig. S3 GO functional enrichment of hBMECs at 12 hpi. **a** Enriched GO terms of hBMECs organized in a network map with edges connecting overlapping gene sets. **b** Ridgeplot visualizing the expression distributions of core enriched genes for the top 30 significant enriched GO terms of hBMECs following A/WSN/33 (H1N1) infection, it also interprets the up and regulated GO terms.

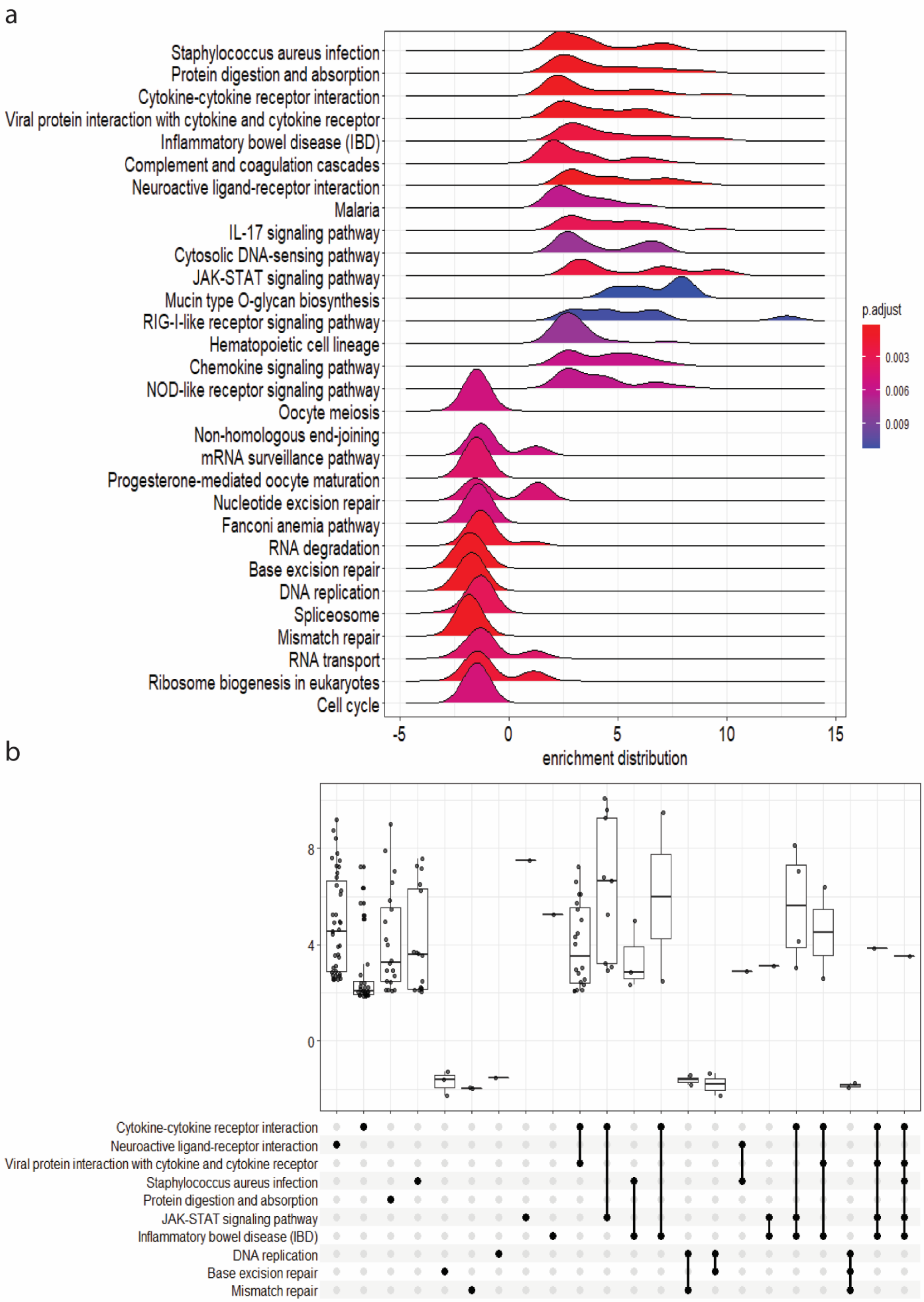
a
Staphylococcus aureus infection
Protein digestion and absorption
Cytokine-cytokine receptor interaction
Viral protein interaction with cytokine and cytokine receptor
Inflammatory bowel disease (IBD)
Complement and coagulation cascades
Neuroactive ligand-receptor interaction
Malaria
IL-17 signaling pathway
Cytosolic DNA-sensing pathway
JAK-STAT signaling pathway
Mucin type O-glycan biosynthesis
RIG-I-like receptor signaling pathway
Hematopoietic cell lineage
Chemokine signaling pathway
NOD-like receptor signaling pathway
Oocyte meiosis
Non-homologous end-joining
mRNA surveillance pathway
Progesterone-mediated oocyte maturation
Nucleotide excision repair
Fanconi anemia pathway
RNA degradation
Base excision repair
DNA replication
Spliceosome
Mismatch repair
RNA transport
Ribosome biogenesis in eukaryotes
Cell cycle
-5
0
5
10
15
enrichment distribution
p.adjust
0.003
0.006
0.009
b
8
4
0
Cytokine-cytokine receptor interaction
Neuroactive ligand-receptor interaction
Viral protein interaction with cytokine and cytokine receptor
Staphylococcus aureus infection
Protein digestion and absorption
JAK-STAT signaling pathway
Inflammatory bowel disease (IBD)
DNA replication
Base excision repair
Mismatch repair

Fig. S4 KEGG functional enrichment of hBMECs 12 hpi a Ridgeplot KEGG based, visualizing the expression distributions of core enriched genes for GSEA enriched KEGG pathways of hBMECs following the virus infection. The plot determines the up and down-regulated pathways. b Upset plot visualizing the overlapped genes among different gene sets.

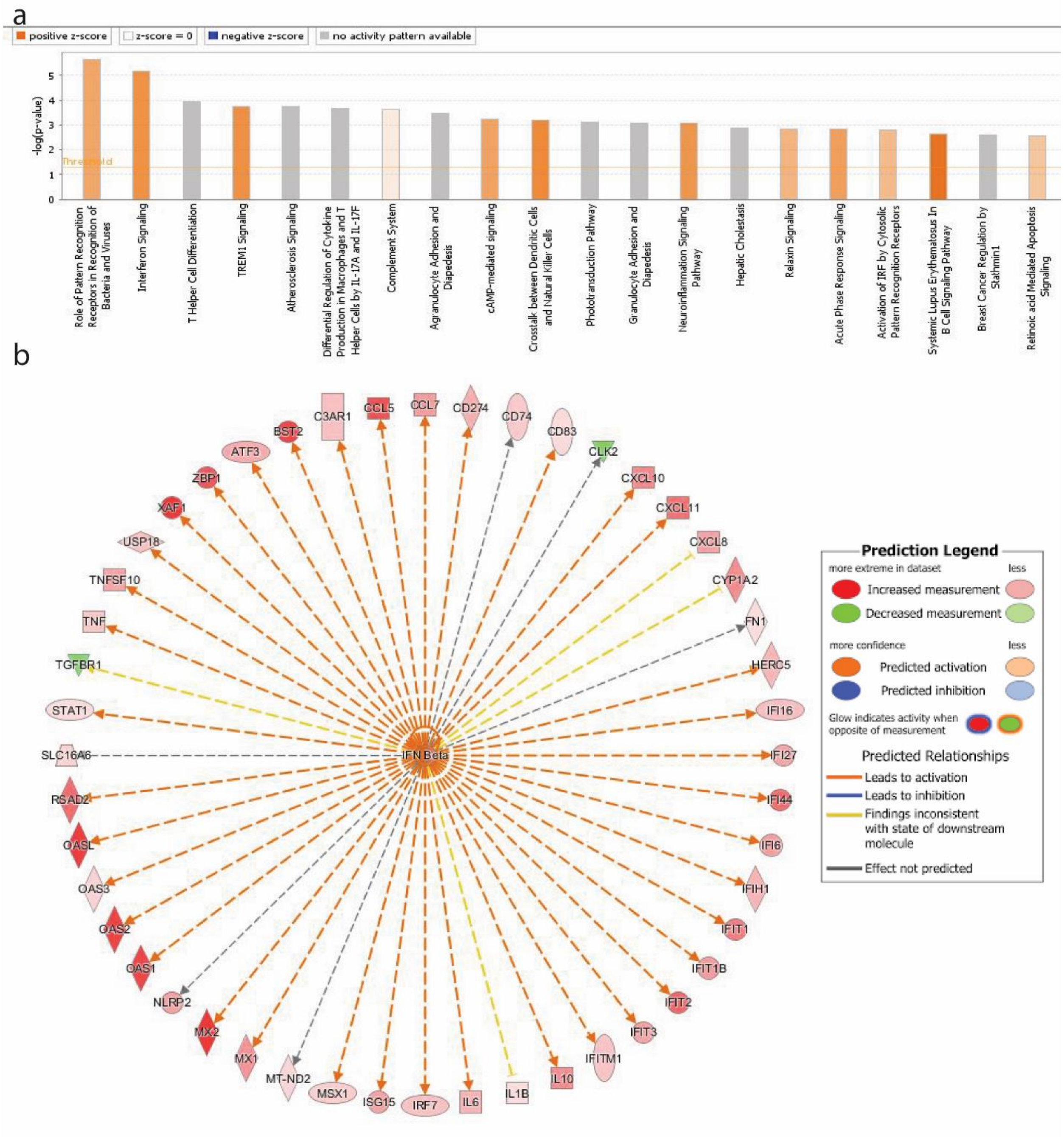

Fig. S5 IPA Pathways and network analysis. a top 20 significant canonical pathways enriched based on the DEGs list uploaded to the IPA for hBMECs 12 hpi. “Role of pattern recognition receptors in recognition of

bacteria and viruses" and "Interferon signaling" are the top two pathways enriched. The threshold indicates a minimum significance level –log (p-value) from Fisher's exact test. The ratio refers to the number of molecules from the dataset that map to the pathway listed divided by the total number of molecules that define the canonical pathway from within the IPA knowledgebase. b The network explains the interaction between IFN-β as an upstream regulator and its target genes.

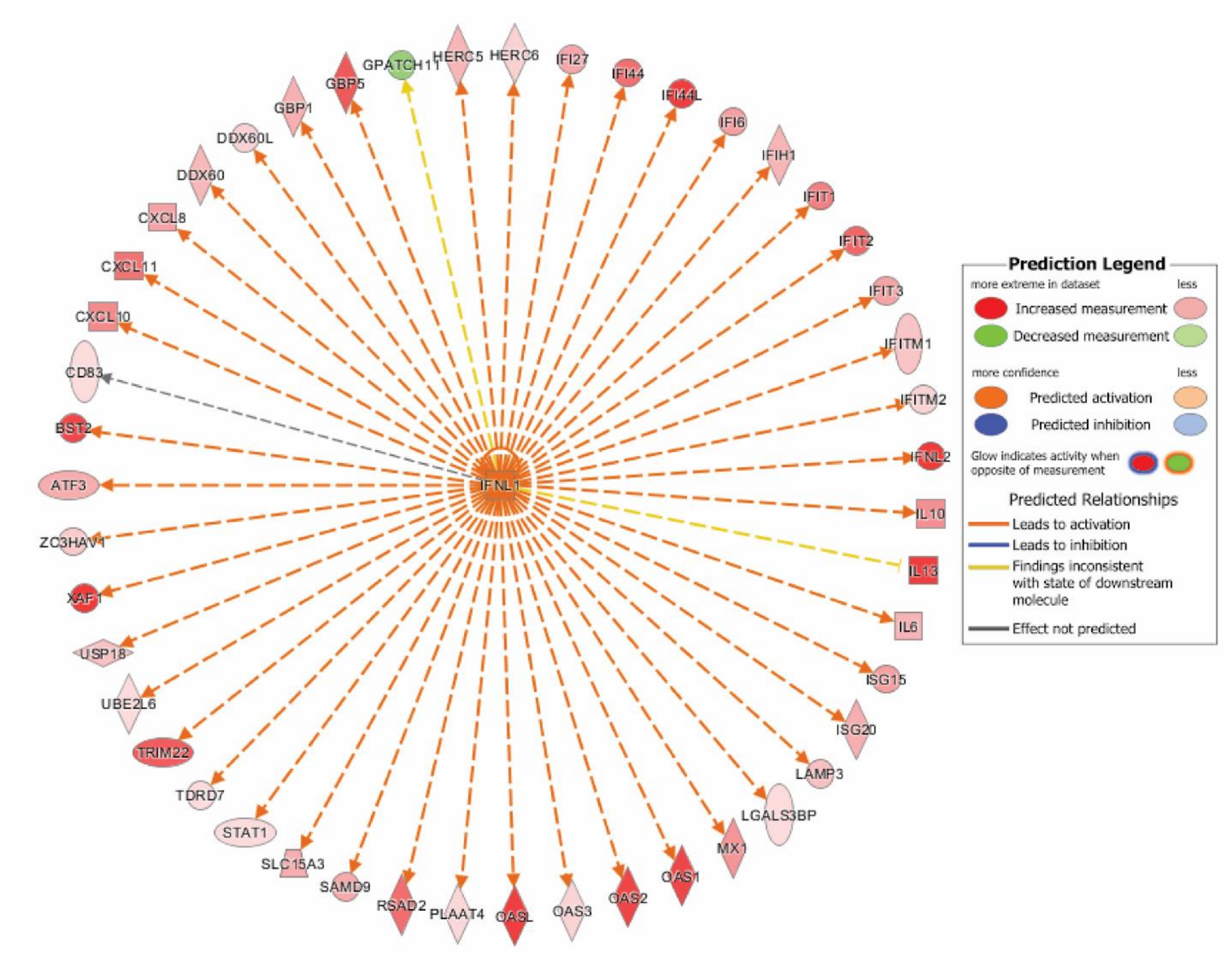

Fig. 6S Regulatory network explaining the interaction between IFNL1 as an upstream regulator and its target genes.

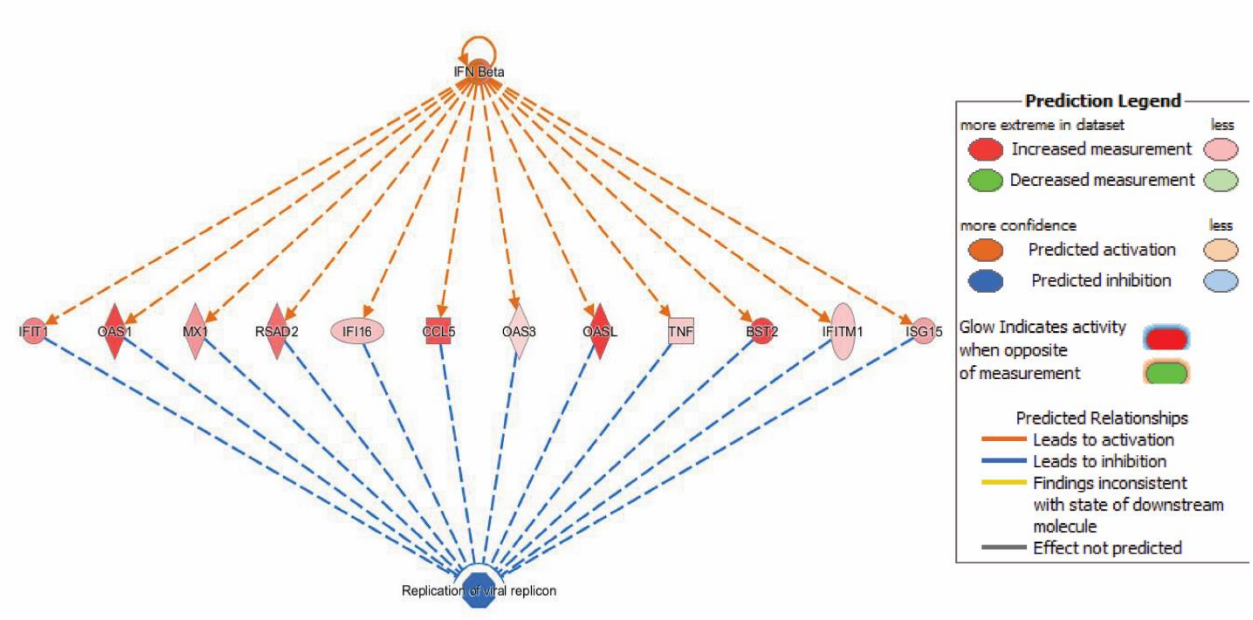

Fig. S7 IPA regulator effects of IFN-β. Integrated results from IFN-β upstream regulator and its downstream effects indicate a potential inhibition for the "virus replication" in hBMECs following 12 h of A/WSN/33 infection. The regulator effects algorithm generates hypotheses that explain how the activation or inhibition of regulators leads to an increase or decrease of function.

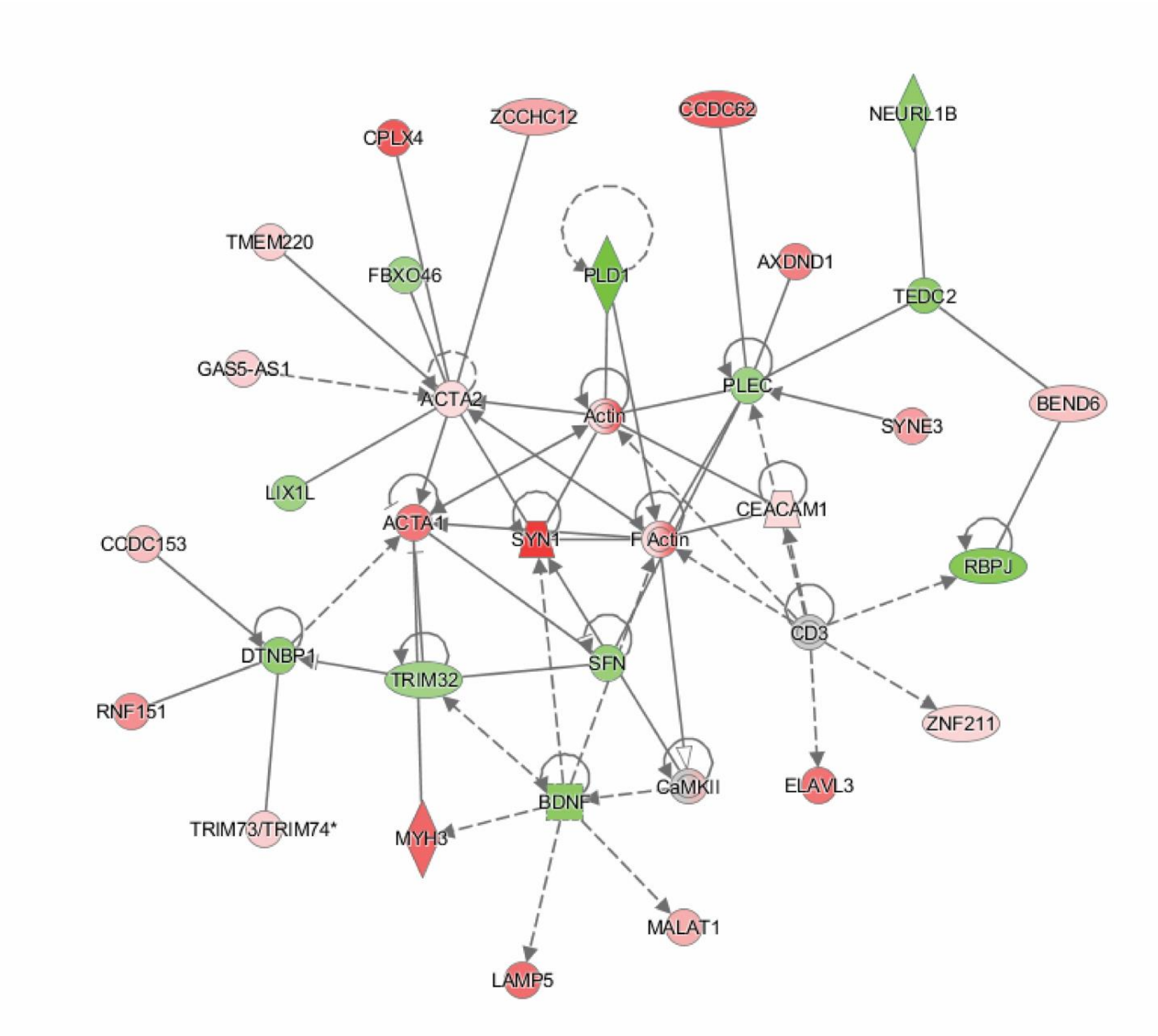

Fig. S8. IPA network analysis. The network reveals the molecules interacting together and functioning in the central nervous system development, Red indicates upregulation; green indicates downregulation.

Table S1 List of primer sequences used for qPCR during the research study.

Primer Name	Primer Sequence
**Viral RNA NP**	GGCCGTCATGGTGGCGAATGAATGGACGGAGAACAAGGATTGC
**NP vRNA-FW**	CTCAATATGAGTGCAGACCGTGCT
**NP vRNA-RV**	GGCCGTCATGGTGGCGAAT
**NDUFS2-FW**	TACCAAGTTCCTCCAGGAGCCA
**NDUFS2-RV**	GGCAAAACCAGGAGCCTTGATC
**SDHA-FW**	GAGATGTGGTGTCTCGGTCCAT
**SDHA-RV**	GCTGTCTCTGAAATGCCAGGCA
**CASP14-FW**	GGTGGATGTGTTCACGAAGAGG
**CASP14-RV**	CCTTCTTGAACCAGCTCTGCTTC
**UQCRH-FW**	CAACAGTGAGAGAGCAATGCGAG
**UQCRH-RV**	CCTCCGTGCAATCCTCTTCTGT
**UQCRFS1-FW**	CCTGTGTTGGACCTGAAGCGG
**UQCRFS1-RV**	CAGAGAAGTCAGGCACCTTGATG
**ISG15-FW**	CTCTGAGCATCCTGGTGAGGAA
**ISG-15-RV**	AAGGTCAGCCAGAACAGGTCGT
**IFNβ-FW**	GACGCCGCATTGACCATCTA
**IFNβ-RV**	TTGGCCTTCAGGTAATGCAGAA
**IFN λ2,3-FW**	CTGACGCTGAAGGTTCTGGAG
**IFN λ2,3-RV**	CGGAAGAGGTTGAAGGTGACAG

This table shows the primers used for the RNAseq results validation by qPCR
